# A 16q22.1 variant confers susceptibility to colorectal cancer as a distal regulator of ZFP90

**DOI:** 10.1038/s41388-019-1055-4

**Published:** 2019-10-22

**Authors:** Chen-Yang Yu, Ji-Xuan Han, Junfang Zhang, Penglei Jiang, Chaoqin Shen, Fangfang Guo, Jiayin Tang, Tingting Yan, Xianglong Tian, Xiaoqiang Zhu, Dan Ma, Ye Hu, Yuanhong Xie, Wan Du, Ming Zhong, Jinxian Chen, Qiang Liu, Danfeng Sun, Yingxuan Chen, Weiping Zou, Jie Hong, Haoyan Chen, Jing-Yuan Fang

**Affiliations:** 1 State Key Laboratory for Oncogenes and Related Genes, Key Laboratory of Gastroenterology and Hepatology, Ministry of Health, Division of Gastroenterology and Hepatology, Shanghai Institute of Digestive Disease, Renji Hospital, School of Medicine, Shanghai Jiao Tong University, 145 Middle Shandong Road, 200001 Shanghai, China; 2 Key Laboratory of Aquacultural Resources and Utilization, Ministry of Education, College of Fishery and Life Science, Shanghai Ocean University, 201306 Shanghai, China; 3 Departments of Surgery and Pathology, Center of Excellence for Cancer Immunology and Immunotherapy, the University of Michigan Rogel Cancer Center, Graduate programs in Immunology and Cancer Biology, University of Michigan School of Medicine, Ann Arbor, MI 48109 USA; 4 Division of Gastrointestinal Surgery, Renji Hospital, School of Medicine, Shanghai Jiao Tong University, 145 Middle Shandong Road, 200001 Shanghai, China; 5 Department of Pathology, Renji Hospital, School of Medicine, Shanghai Jiao Tong University, 145 Middle Shandong Road, 200001 Shanghai, China

**Keywords:** Colorectal cancer, Cancer genetics, Mechanisms of disease

## Abstract

Genome-wide association studies (GWASs) implicate 16q22.1 locus in risk for colorectal cancer (CRC). However, the underlying oncogenic mechanisms remain unknown. Here, through comprehensive filtration, we prioritized rs7198799, a common SNP in the second intron of the CDH1, as the putative causal variant. In addition, we found an association of CRC-risk allele C of rs7198799 with elevated transcript level of biological plausible candidate gene ZFP90 via expression quantitative trait loci analysis. Mechanistically, causal variant rs7198799 resides in an enhancer element and remotely regulate ZFP90 expression by targeting the transcription factor NFATC2. Remarkably, CRISPR/Cas9-guided single-nucleotide editing demonstrated the direct effect of rs7198799 on ZFP90 expression and CRC cellular malignant phenotype. Furthermore, ZFP90 affects several oncogenic pathways, including BMP4, and promotes carcinogenesis in patients and in animal models with ZFP90 specific genetic manipulation. Taken together, these findings reveal a risk SNP-mediated long-range regulation on the NFATC2-ZFP90-BMP4 pathway underlying the initiation of CRC.

## Introduction

Colorectal cancer (CRC) ranks as the third most common cancer and the fourth leading cause of cancer-related death globally [1–3]. Inherited susceptibility is a major component of CRC predisposition with an estimated 12–35% of risk attributing to genetic factors [4, 5]. Revolutionary genome-wide association studies (GWAS) and subsequent fine-mapping research have positioned over 50 genetic susceptibility loci of CRC in both European and Asian populations [6]. However, most of the identified CRC genetic variants are tag single-nucleotide polymorphisms (tag SNPs), residing in intergenic and intronic regions with unknown function. Thus, one of the major challenges in the post-GWAS era is to identify the causal genetic variant(s) that accounts for the biological phenotype linked to specific diseases [7–9].

Both empirical and computational data supports the notion that a considerable proportion of trait-associated loci will harbor variants that influence the abundance of specific gene transcripts. These variants are often referred to as expression quantitative trait loci (eQTLs) [10]. Several landmark studies have unequivocally shown that many transcripts in the human genome are influenced by inherited variations [11–13]. Studies on the associations between genetic variation and gene expression offer a way to connect the risk variants to their putative target genes or transcripts [9]. 16q22.1 region is associated with CRC development in multiple populations [14–16]. Carvajal-Carmona et al. sought to fine-map the location of the functional variants for 16q22.1 region (CDH1/CDH3) [17]. Interestingly, the expression quantitative trait locus (eQTLs) analysis on peripheral blood cells has shown a number of highly associated SNP alleles in 16q22.1 region, which correlated with mRNA levels of Zinc-finger protein 90 (ZFP90) in the long distance, but not with the flanking gene CDH1 or CDH3 [17]. However, to date there are no known mechanisms underlying its function.

In the current study, we report the identification of the rs7198799 variant as the functional basis defining the association between 16q22.1 and CRC. We demonstrate that rs7198799 controls ZFP90 expression by targeting the transcriptional factor NFATC2 and by remotely acting as an enhancer for ZFP90. Furthermore, the oncogenic role of ZFP90 has been validated in CRC patients and in carcinogen-induced CRC models with ZFP90-deficiency mice. Thus, our study has identified the causal variant in 16q22.1 locus and the downstream NFATC2-ZFP90-BMP4 pathway, with a biological, mechanistic, and clinical impact on CRC development.

## Results

### CRC-risk haplotype at 16q22.1 interacts with ZFP90 in colon epithelium

16q22.1 region, is one of the strongest GWAS signals of CRC [15]. This region contains ~534 common variants, including the CRC-associated tag SNP, rs9929218 (chr16: 68787043, hg38), of which 131 SNPs were in high linkage disequilibrium (LD) in Europeans (*r*^2^ ≥ 0.8) (Fig. S1a). LD is the nonrandom association of alleles at linked loci. The GWAS study takes advantage of the fact that LD may exist between a known lead SNP and an unknown trait locus not directly genotyped. The tag SNPs (here is rs9929218), identified by GWAS study, are informative but often not causative. The causative SNP may lie anywhere within the high LD block surrounding the tag SNP, probably one in 131 common variants. These variants were also proved to be strongest association signals by fine-mapping on 16q22.1 locus conducted in six European cohorts [17] (Fig. S1a). Owing to these variants lie within a narrow region (chr16: 68668174—68802588, hg38), the identification of the likely causal variant becomes very challenging.

**Fig. 1:**
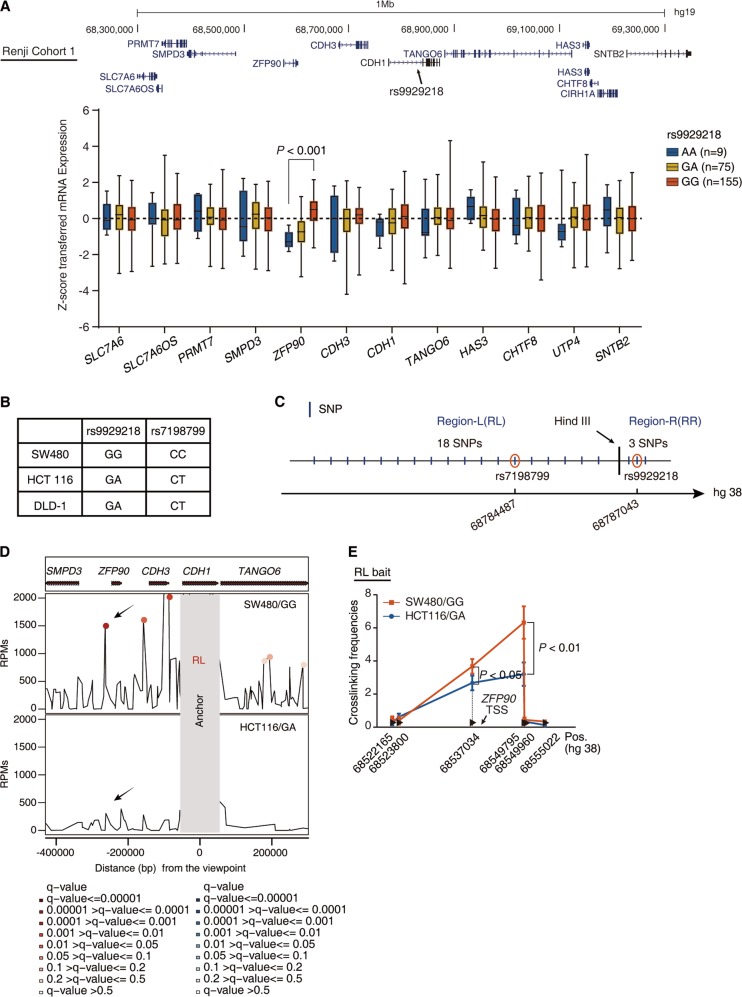
CRC-risk haplotype at 16q22.1 interacts with ZFP90 in colon epithelium. **a** Real-time PCR was performed to determine the mRNA levels of *ZFP90* and 11 nearby genes within 1 Mb window in normal colorectal mucosa in Cohort 1. Results are plotted according to genotype at rs9929218. *P* value was calculated by linear regression model. **b** Sanger sequencing of genotypes of SNP-rs9929218-G/A and SNP-rs7198799-C/T in three colorectal cancer cell lines. **c** Eighteen SNPs in the same haplotype with SNP-rs9929218 with LD > 0.8 located in RL. Three SNPs (containing SNP-rs9929218 itself) in the same haplotype with SNP-rs9929218 with LD > 0.8 located in RR. RL and RR were separated by Hind III recognition sequence. **d** 4C was used to identify the chromatin interactions between RL (region left) and the ZFP90 region in HCT116 and SW480 cells. RL served as anchor. **e** 3C-qPCR was performed to determine the relative interaction frequencies between RL (containing SNP-rs7198799) and *ZFP90* promoter region, comparing the relative abundance of ligation products formed between the fragment mapping to RL and each of the target fragments in *ZFP90* promoter region. Results are normalized to the relative abundance of control region. *n* = 3 with triplicates, non-paired two-tailed *t*-test, SW480 vs HCT116

The availability of eQTL information could provide immediate insight into a biological basis for disease associations identified through GWAS studies, and can help to identify networks of genes involved in disease pathogenesis [18]. Therefore, we isolated colorectal mucosa which is mainly composed of intestinal epithelial cells (IEC) in 239 normal colorectal tissue samples collected from Renji Hospital (Cohort 1) (Table S1). To predict putative target genes of rs9929218, we examined its location and performed eQTL analysis with the genes surrounding CDH1. We identified twelve candidate genes within 500 kb upstream and downstream of tag SNP rs9929218 (Figs. 1a and S1b). *Cis*-eQTL analysis revealed that rs9929218 significantly correlated with the transcript levels of ZFP90, but not with other eleven proximal genes (Fig. 1a).

As mentioned above, GWAS studies may identify CRC-risk variants, but are incapable of determining the causative SNPs on their own [8]. Given that ZFP90, the potential candidate target gene of rs9929218, resides over 247 kb away from the tag SNP region, the regulatory mechanism of ZFP90 is unknown. To examine whether there was a direct chromatin interaction between the risk SNP region and ZFP90 promoter region, we first performed circular chromosome conformation capture (4C) on SW480 cell line because it carried two risk haplotypes (Fig. 1b).

When anchored at the ZFP90 promoter, the highest peak was detected within the risk SNP region of 16q22.1 (Fig. S1c). To explore the causative SNP, the risk SNP region located in the highest peak at chr16: 68778398 to 68787223 containing 21 SNPs was divided into two segments, namely region left (RL, containing 18 SNPs) and region right (RR, containing 3 SNPs), according to the restriction endonuclease (Hind III) cutting recognition site(Fig. 1c). Using RL as a view point in 4C analysis, a significant DNA-DNA interaction was observed in ZFP90 promoter region (Fig. 1d upper panel), whereas no significant signal was detected when RR was used as a view point (Fig. S1d upper panel). Quantitative chromosome conformation capture assays (3C-qPCR) validated these findings (Fig. 1e and Fig. S1e, f). To further exclude the possibility that ZFP90 expression was regulated by RR via enhancer–promoter looping, we established RR-deleted (ΔRR) HCT116 cells. As expected, ZFP90 expression was unchanged in RR-deleted HCT116 (Fig. S1g). Taken together, the causative variant of 16q22.1 region may be located at the RL region containing 18 SNPs and target ZFP90.

### SNP-rs7198799 is a causal variant constituting a ZFP90 distal enhancer

**Fig. 2:**
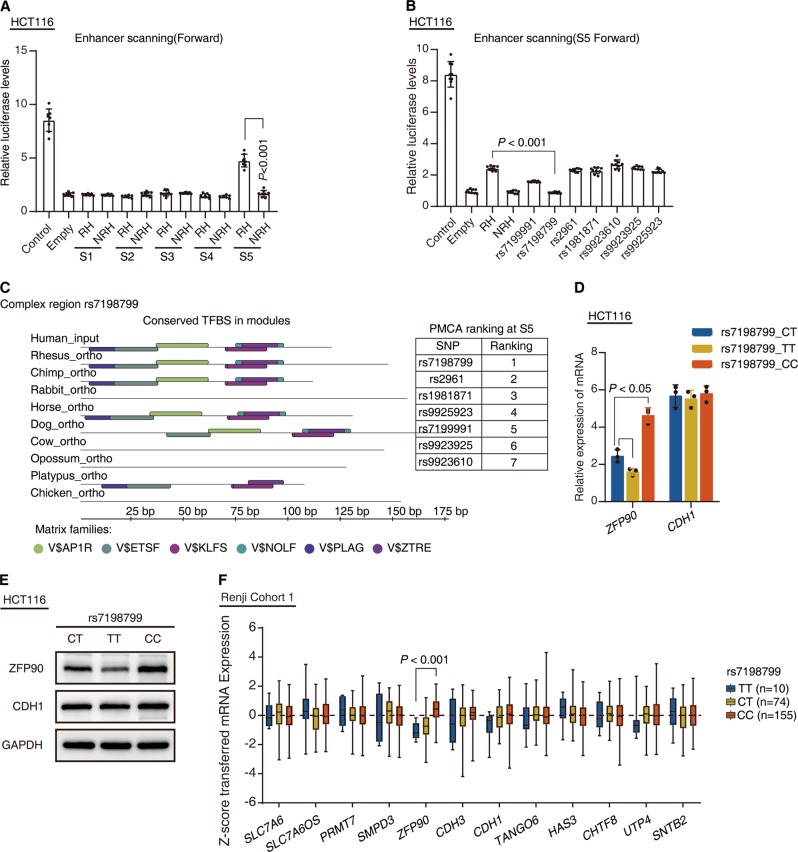
SNP-rs7198799 is a causal variant constituting a *ZFP90* distal enhancer. **a** Luciferase reporter assay was performed to detect the enhancer activity of each segment with the risk and nonrisk haplotypes. *n* = 3 with eight replicates, non-paired *t*-test, RH vs NRH. RH risk haplotype, NRH nonrisk haplotype. **b** Luciferase reporter assay was performed to detect the enhancer activity. Each SNP was mutated from the risk allele to the nonrisk allele in HCT116 cells. *n* = 3 with eight replicates, non-paired two-tailed *t*-test, S5-RH carrying rs7198799 nonrisk allele vs S5-RH carrying rs7198799 risk allele. **c** PMCA analyses evolutionary conservation of *cis*-regulatory modules across related species in seven SNPs with their flanking sequences. PMCA scores of each SNP was calculated and ranked in the table. **d** Real-time PCR was performed to determine the expression change in mRNA levels of *ZFP90* and *CDH1* with SNP-rs7198799 mutation (CT > TT/CC). *n* = 3 with triplicates, non-paired two-tailed *t*-test, rs7198799_CT HCT116 vs rs7198799_TT or rs7198799_CC HCT116. **e** Western blot was performed to determine expression change in protein levels of *ZFP90* and *CDH1* with SNP-rs7198799 mutation (CT > TT/CC). **f** Real-time PCR was performed to determine the mRNA levels of *ZFP90* and 11 nearby genes within 1 Mb in normal colorectal mucosa in Cohort 1. Results are plotted according to genotype at rs7198799. *P* value was calculated by linear regression model

It is generally thought that the functional SNPs can modify the activity of transcriptional regulatory regions, such as enhancers, that interact with the promoter of target genes [9, 19]. Enhancer RNAs are considered the predictors of active enhancers [20, 21]. In support of the possibility, we found that the genomic region containing RL was an intestinal epithelial cell specific enhancer in FANTOM5 databases (Fig. S2a). Combined with 4C results mentioned above, we hypothesized that the RL of the risk SNP region may execute potential enhancer function. To test this possibility, a luciferase assay was conducted to systematically scan the RL genomic region [19]. The RL region was evenly divided into five parts, namely, segment 1 (S1), S2, S3, S4, and S5. The luciferase assay revealed that S5, carrying seven SNPs, had higher enhancer activities than four other segments in HCT116 and DLD1 cell lines (Figs. 2a and S2b, c). This effect was orientation specific (Fig. S2d, e). We also observed higher luciferase activities in the vector carrying risk haplotype of S5 compared with nonrisk haplotype (Figs. 2a and S2c). To probe whether S5 carried the causal variant, we performed CRISPR/Cas9 genome-editing experiments in HCT116. Five sgRNAs were designed to specifically target S5. Unfortunately, we technically failed to obtain positive clones with specific and exclusive S5 deletion. Alternatively, we successfully deleted a 4632 bp region including S3–S5 (Fig. S2f). S2-deleted (ΔS2) HCT116 cells were established to be a control. Real-time PCR and western blot assays revealed that loss of S3–S5 resulted in reduced ZFP90 expression, but had no effect on CDH1 expression, compared with wild-type (WT) cell lines (Fig. S2g, h). In the meanwhile, S2 deletion did not affect ZFP90 expression (Fig. S2i).

S5 contains seven SNPs: rs7199991, rs7198799, rs2961, rs1981871, rs9923610, rs9923925, and rs9925923 (Fig. S2b). To identify the causative variant among the seven candidate SNPs, these SNPs were individually mutated from risk haplotype of S5 to nonrisk alleles. There was a strong decrease in enhancer activity for the vector carrying rs7198799 from C (risk allele) to T (nonrisk allele), but not the other six SNPs (Figs. 2b and S2j). In addition, phylogenetic module complexity analysis (PMCA) [22] was performed to test the causal variant possibility. The highest PMCA score was also found in the rs7198799 region in S5 (Fig. 2c). To investigate whether rs7198799 was directly involved in the regulation of ZFP90 expression, we converted the genotype of rs7198799 from genotype CT to TT and CT to CC in HCT116 cell line with CRISPR/Cas9-mediated genome-editing approach (Fig. S2k). The mutated cells with rs7198799/TT expressed markedly lower transcriptional levels of ZFP90 but not CDH1, compared with the parental cells (Fig. 2d, e). On the other hand, rs7198799/CC expressed markedly higher transcriptional levels of ZFP90 (Fig. 2d, e). Moreover, ZFP90 expression was not affected in the negative control, the mutated HCT116 cell line with rs7199991/GG converted from wild-type HCT116 with rs7199991/TG (Fig. S2l, m). Previous 4C assay revealed that the interaction between rs7198799 and ZFP90 promoter was enriched in SW480 cell line, but remarkably decreased in HCT116. This is probably due to SW480 and HCT116 cell lines, respectively, carried two copies and one copy of risk alleles on rs7198799 (Fig. 1b, d lower panel, e and Fig. S1d lower panel, e, f). Moreover, *Cis*-eQTL analysis on Renji Cohort 1 confirmed that rs7198799 significantly correlated with the transcript levels of ZFP90, but not with other eleven proximal genes (Figs. 2f and S2n). These data suggest that the transcriptional regulation of ZFP90 expression is allele-dependent and rs7198799 is the causative variant of 16q22.1.

### Differential activity of rs7198799 is mediated by NFATC2

**Fig. 3:**
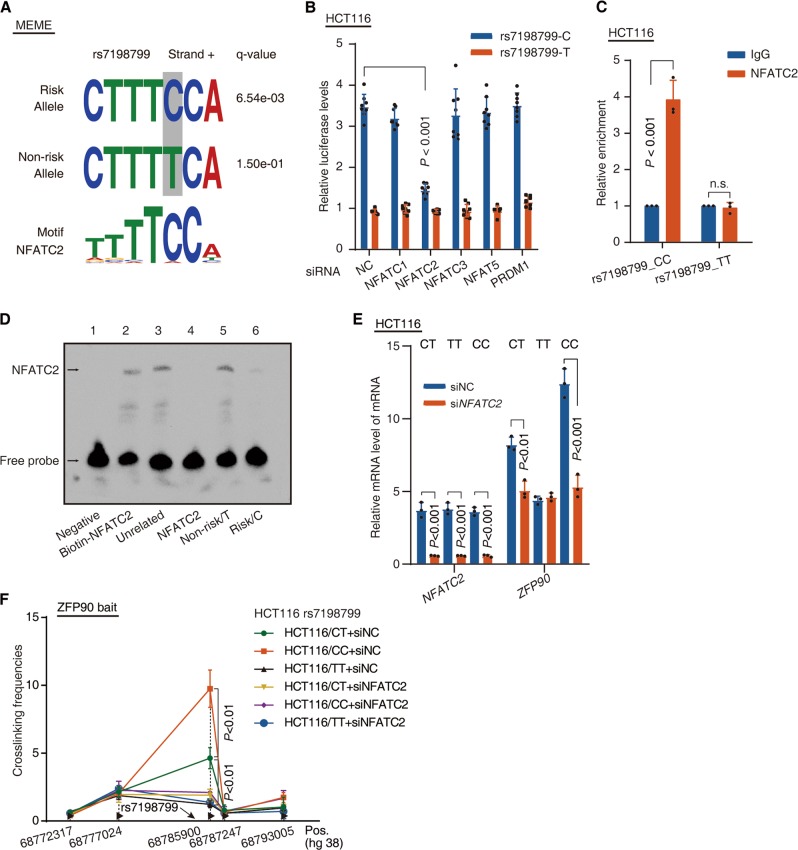
Differential activity of rs7198799 is mediated by NFATC2. **a** rs7198799 resides within NFATC2 DNA-binding motifs. Binding affinity was calculated by MEME SUITE algorithm. **b** Luciferase-based enhancer assays was performed to detect changes of enhancer activity. HCT116 cells were transfected with LRF vector containing rs7198799-centered region with allele C or T and siRNA. *n* = 3 with eight replicates, non-paired two-tailed *t*-test. **c** Real-time PCR of the ChIP samples was performed to determine NFATC2 binding efficiency to SNP-rs7198799 in rs7198799_TT and rs7198799_CC HCT116 cells. *n* = 3 with triplicates, non-paired two-tailed *t*-test, IgG vs NFATC2 Ab. **d** EMSA was performed to determine the binding affinity between NFATC2 and SNP-rs7198799 sequence. Lane 1 represents negative control. The binding of NFATC2 consensus sequence (lane 2) is competed by an unrelated sequence (lane 3), the NFATC2 consensus sequence (lane 4), and the sequence containing the rs7198799 T allele (lane 5) and C allele (lane 6). **e** HCT116 cells (rs7198799_CT, rs7198799_TT, or rs7198799_CC) were transfected with *NFATC2* siRNA or negative control (NC) siRNA, respectively. Real-time PCR was performed to determine the mRNA levels of *NFATC2* and *ZFP90*. **f** 3C-qPCR was performed to determine the relative interaction frequencies between *ZFP90* promoter region and rs7198799 region, comparing the relative abundance of ligation products formed between the fragment mapping to *ZFP90* promoter region and each of the target fragments in rs7198799 region. HCT116 cells (rs7198799_CT, rs7198799_TT, or rs7198799_CC) were transfected with *NFATC2* siRNA or NC siRNA, respectively Results are normalized to the relative abundance of control region. *n* = 3 with triplicates, non-paired two-tailed *t*-test

Given that SNP-specific changes are thought to modify enhancer activity by altering transcription factor (TF) binding [23, 24], we next examined whether rs7198799 directly alters the DNA-binding motif by Genomatix SNPInspector software. This analysis indicated that rs7198799 overlaps with binding motif of NFAT family. Notably, NFATC2 has a higher preference for the risk allele C (Figs. 3a and S3a). Similarly, rs7198799 overlaps with a predicted PRDM1 motif with a higher preference for the nonrisk allele T (Fig. S3a). To investigate whether NFAT family and PRDM1 are involved in a potential regulation of enhancer activity of rs7198799, we performed luciferase-based enhancer assays combined with knockdown of these TFs in HCT116 cells. We observed that knockdown of NFATC2 resulted in a remarkably decrease of the enhancer activity of rs7198799 region with allele C but not allele T (Fig. 3b). Moreover, ChIP-qPCR results also showed the enrichment of NFATC2 at rs7198799-containing region with allele C but not with allele T in SNP-editing HCT116 cell lines (Fig. 3c). To investigate whether the potential causative SNP-rs7198799 would affect the binding affinity of NFATC2 in an allele-dependent manner, we conducted an electrophoretic mobility shift assay (EMSA) using the nuclear extract from HCT116 cells. EMSA results indicated that an oligonucleotide corresponding to a C allele of SNP-rs7198799 exhibited stronger binding affinity to NFATC2 than that to a T allele (Fig. 3d).

To evaluate whether ZFP90 expression influenced by its distal enhancer containing rs7198799 depends on NFATC2, we conducted NFATC2 knockdown in HCT116 cell lines with different genotypes (CT, CC, and TT) at rs7198799. We observed that the difference of ZFP90 expression among three isogenic HCT116 cell lines diminished after NFATC2 knockdown (Fig. 3e). We next performed 3C experiments in the three isogenic HCT116 cells, and found that the CC genotype HCT116 cells had higher cross-linking frequencies between rs7198799 enhancer and ZFP90 promoter than parental and TT cells. Moreover, NFATC2 knockdown had a large impact on observed interaction between rs7198799 enhancer and ZFP90 (Fig. 3f). Thus, these results revealed that rs7198799 is the causative variant in 16q22.1 region and may target ZFP90 expression through NFATC2-mediated transcription (Fig. S3b).

### ZFP90 affects colorectal tumorigenesis in vitro and in vivo

**Fig. 4:**
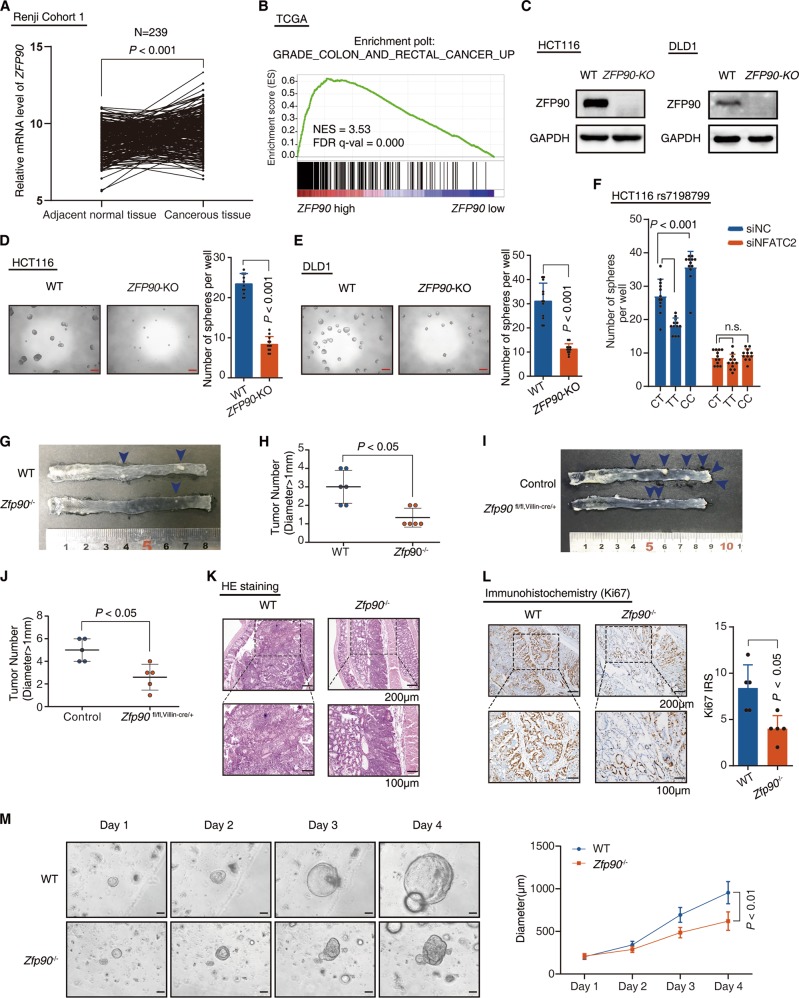
ZFP90 affects colorectal tumorigenesis in vitro and in vivo. **a** Real-time PCR was performed to determine the mRNA levels of *ZFP90* in cancerous colorectal tissue and normal adjacent colorectal tissue. Cohort 1, paired two-tailed Wilcoxon test, adjacent normal mucosa vs cancerous tissue. **b** GSEA was used to identify the differential gene profiles between ZFP90-high CRC tumors and ZFP90-low CRC tumors in TCGA datasets. NES normalized enrichment score. **c** Western blot was performed to determine the protein level of ZFP90 in HCT116 and DLD1 cells (WT and *ZFP90*-KO). Tumor sphere assay was performed with HCT116 (**d**) and DLD1 (**e**) cells (WT or *ZFP90*-KO). Scale bars represent 100 μm. *n* = 12, nonparametric two-tailed Mann–Whitney test, WT vs *ZFP90*-KO. **f** Tumor sphere assay was performed with HCT116 cells (rs7198799_CT, rs7198799_TT, or rs7198799_CC). The cells were treated with *NFATC2* siRNA or NC siRNA. Scale bars represent 100 μm. *n* = 12, non-paired two-tailed *t*-test, CT vs TT, CT vs CC. **g** Representative image of colonic tumors (Blue arrows) in WT mice and *Zfp90*^−/−^ mice. **h** Tumor numbers for WT mice and *Zfp90*^−/−^ mice. *n* = 6, Nonparametric two-tailed Mann–Whitney test, WT vs *Zfp90*^−/−^. **i** Representative image of colonic tumors (Blue arrows) in Control mice and *Zfp90*^fl/fl, Villin-cre/+^ mice. **j** Tumor numbers for Control mice and *Zfp90*^fl/fl, Villin-cre/+^ mice. *n* = 5, Nonparametric two-tailed Mann–Whitney test, Control vs *Zfp90*^fl/fl, Villin-cre/+^. **k** Representative histological staining of tumors from WT mice and *Zfp90*^−/−^ mice (H&E staining). **l** Representative immunohistochemical staining of Ki67 protein in tumors from WT mice and *Zfp90*^−/−^ mice. Immunoreactive score of Remmele and Stegner (IRS) of Ki67 protein was quantitated. *n* = 5, Nonparametric two-tailed Mann–Whitney test, WT vs *Zfp90*^−/−^. **m** Representative images of cancer colonoids derived from WT mice and *Zfp90*^−/−^ mice. Scale bars represent 200 μm. Quantitated result was performed to indicate cancer colonoids growth. *n* = 20, non-paired two-tailed *t*-test, WT vs *ZFP90*^−/−^

ZFP90 is a transcription repressor with its role in CRC progression poorly characterized. Thus, we evaluated ZFP90 expression and its clinical relevance in CRC patients. ZFP90 expression was significantly increased in CRC tumor tissues from patients compared with nontumor tissues in Renji cohorts and CRC TCGA datasets (Figs. 4a, S4a, b, c and Table S2). Interestingly, gene set enrichment analysis (GSEA) revealed that the gene sets related to Grade_Colon_And_Rectal_Cancer_Up and Sabates_Colorectal_Adenoma_Up positively correlated with ZFP90 high expression in TCGA CRC datasets (Figs. 4b and S4d). We next evaluated and compared ZFP90 expression with different clinicopathologic features in Renji Cohort 2. We found that ZFP90 expression positively correlated with pathological grade and AJCC stage (Fig. S4e). The Kaplan–Meier analyses showed that high ZFP90 expression was associated with a poor prognosis in CRC patients in Cohort 2 (Fig. S4f) and an independent CRC database (Fig. S4g). The data suggested that ZFP90 is a clinical oncogene in patients with CRC.

To investigate the biological role of ZFP90 in CRC, we first measured ZFP90 function on CRC cell tumorigenic potential. Knockout of ZFP90 significantly impaired CRC sphere formation (Fig. 4c, d, e) in HCT116 and DLD1 cells. Next, we injected different amounts of CRC cells into NOD/Shi-*scid*/IL-2Rγ^null^ (NSG) mice, and found knockout of ZFP90 reduced HCT116 tumor formation potential. The same phenomenon was observed in nude mouse models as well (Fig. S4h, i). Interestingly, HCT116 with rs7198799-TT had lower CRC sphere formation ability compared with HCT116 with rs7198799-CT, while HCT116 with rs7198799-CC had higher CRC sphere formation ability (Fig. 4f). After knockdown of NFATC2 expression, the difference of sphere formation ability among these three isogenic cell lines diminished (Fig. 4f). These results suggested that the effect of the SNP-rs7198799 on CRC cell sphere formation ability largely depended on NFATC2 existence.

To identify the role of Zfp90 in an induced CRC model in vivo, Zfp90 gene was specifically deleted in the mouse genome, regarding that Zfp90 transgenic mice is infertile [25]. Using the CRISPR/Cas9 system [26], we deleted the exon2 and exon3 of Zfp90 gene (Fig. S4j), and generated Zfp90-KO mutants (Zfp90^−/−^) in C57BL/6J mice background. We challenged the Zfp90^−/−^ mice and their genetic control siblings with azoxymethane (AOM) to induce CRC development (Fig. S4k). We observed lower tumor numbers (Fig. 4g, h) and smaller tumor sizes (Fig. S4l) in Zfp90^−/−^ mice as compared with wild-type mice. The similar phenomenon was observed in Zfp90^fl/fl, Villin-cre/+^ mice (Figs. 4i, j and S4m), whose Zfp90 was specifically deleted in IEC (Fig. S4n). Histological analysis demonstrated poorly-differentiated CRC in WT mice and moderately-differentiated CRC in Zfp90^−/−^ mice (Fig. 4k). Furthermore, we detected more Ki67^+^ proliferative tumor cells in WT mice than Zfp90^−/−^ mice (Fig. 4l). The growth and the size of tumor organoids were dramatically reduced in Zfp90^−/−^ mice compared with WT mice (Fig. 4m). As expected, Zfp90^−/−^ mice also experienced longer survival time (Fig. S4o). Thus, intrinsic Zfp90 promotes intestinal tumorigenesis in vivo.

### ZFP90 targets BMP4 to control carcinogenesis

**Fig. 5:**
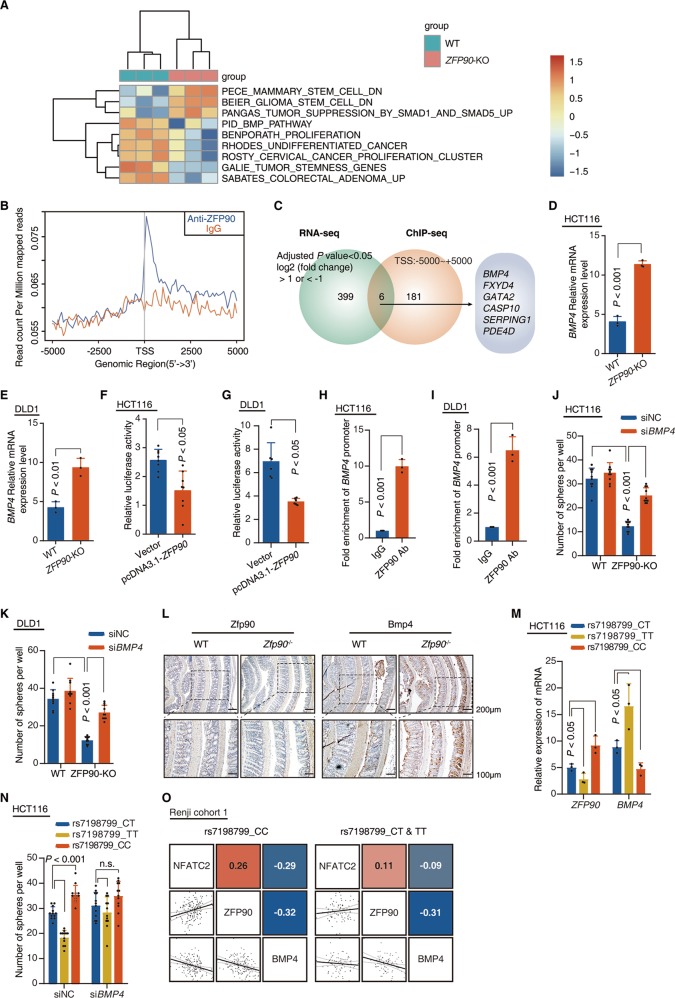
ZFP90 targets BMP4 to control carcinogenesis. **a** ssGSEA analysis was conducted to investigate the relationship between *ZFP90* expression and carcinogenesis-related pathways in HCT116 cells (WT vs *ZFP90*-KO). **b** ChIP-seq profile of ZFP90 global genomic binding at the target sites in HCT116 cells. A 5-kb interval centered on the called ZFP90 peak is shown. **c** Venn diagram shows the gene regulated by *ZFP90* knockout (405 genes), or whose promoter was occupied by ZFP90 (187 genes), or both (6 genes). **d, e** Real-time PCR was performed to determine the mRNA level of *BMP4* in HCT116 and DLD1 cells (WT or *ZFP90*-KO). *n* = 3 with triplicates, non-paired two-tailed *t*-test, WT vs *ZFP90*-KO. **f**, **g** Luciferase activity was measured in HCT116 and DLD1 cells transfected with empty vector (pcDNA3.1) or pcDNA3.1-*ZFP90*. The luciferase reporter vector was generated by inserting the 1 kb promoter fragment of *BMP4* into pGL3-enhancer plasmid. *n* = 3 with eight replicates, non-paired two-tailed *t*-test, empty vector vs pcDNA3.1-*ZFP90*. **h**, **i** Real-time PCR of the ChIP samples was performed to determine ZFP90 binding efficiency to *BMP4* gene promoter in HCT116 and DLD1 cells. *n* = 3 with triplicates, non-paired two-tailed *t*-test, IgG vs ZFP90 Ab. Tumor sphere assay was performed with HCT116 (**j**) and DLD1 (**k**) cells (WT or *ZFP90*-KO). The cells were treated with *BMP4* siRNA or NC siRNA. Scale bars represent 100 μm. *n* = 12, non-paired two-tailed *t*-test. **l** Representative immunohistochemical staining of Zfp90 and Bmp4 proteins in normal colon mucosa of WT mice and *Zfp90*^−/−^ mice. **m** Real-time PCR was performed to determine the mRNA level of *BMP4* in HCT116 cells (rs7198799_CT, rs7198799_TT, or rs7198799_CC). *n* = 3 with triplicates, non-paired two-tailed *t*-test, CT vs TT, CT vs CC. **n** Tumor sphere assay was performed with HCT116 cells (rs7198799_CT, rs7198799_TT, or rs7198799_CC). HCT116 cells were transfected with *BMP4* siRNA or NC siRNA. n = 12, non-paired two-tailed *t*-test, CT vs TT, CT vs CC. **o** Correlations among NFATC2, ZFP90, and BMP4 levels in human normal colorectal mucosa (Cohort 1)

As the clinical implication and biological function of ZFP90 had been clarified above, we performed an RNA-seq analysis and compared the gene expression profiles between HCT116 WT cells and ZFP90-knockout cells to determine the underlying molecular mechanism. Knockout of ZFP90 downregulated 249 gene expressions and upregulated 156 gene expressions (log2(fold_change) > 1 or < −1, FDR *P* value < 0.05, GEO number: GSE121621) (Table S3). Single-sample gene set enrichment analysis (ssGSEA) revealed that the gene sets, which regulated cancer initiation and stemness, were enriched in ZFP90 competent, but not in ZFP90 deficient CRC cells (Fig. 5a). We next initially performed a ChIP coupled with high-throughput sequencing (ChIP-seq) to determine the localization of ZFP90 genomic binding genome-wide in HCT116 cells. ZFP90 ChIP-Seq data revealed 5549 called peaks with 187 gene promoters occupied by ZFP90 (Figs. 5b and S5a). The GO pathway analysis [27] showed that the target genes of ZFP90 were associated with cell proliferation and pathways in cancer (Fig. S5b). Next, we combined the RNA-seq data mentioned above to determine the relationship between ZFP90 binding and ZFP90-mediated changes in the gene expression profiles. Overlapping analysis found that among the 187 genes, six genes (BMP4, FXTD4, GATA2, CASP10, SERPING1, and PDE4D) were also dysregulated in ZFP90 knockout cells (Fig. 5c and Table S4). The real-time PCR (Fig. S5c) and ChIP-qPCR assay (Fig. S5d) showed that BMP4 and GATA2 were the two genes directly regulated by ZFP90. Given that GATA2 has been reported as an oncogene [28], GATA2 expression was increased in ZFP90 knockout HCT116 cells (Fig. S5c), and ZFP90 knockout blocked colorectal carcinogenesis, we chose BMP4 as the biological candidate target of ZFP90 for validation. Real-time PCR (Fig. 5d) demonstrated that BMP4 expression was significantly increased in ZFP90-KO HCT116 cells compared with controls. Similar results were observed in DLD1 cells (Fig. 5e). Furthermore, the luciferase assay revealed that overexpression of ZFP90 impaired the transcriptional level of the BMP4 promoter in HCT116 cells (Fig. 5f) and DLD1 cells (Fig. 5g). ChIP-qPCR data showed that ZFP90 directly bound to the promoter region of *BMP4* gene in HCT116 (Fig. 5h) and DLD1 cells (Fig. 5i). The data indicated that ZFP90 may negatively regulate BMP4 transcription in CRC cells. Hence, we hypothesized that BMP4 was a key mediator of the biological function of ZFP90 in CRC. As expected, downregulation of BMP4 partly blocked the ZFP90-KO-reduced cell sphere formation in HCT116 cells (Fig. 5j) and DLD1 cells (Fig. 5k). Moreover, ZFP90 expression was negatively correlated with BMP4 in human normal colon mucosa (Fig. 5o) and mouse normal colon mucosa (Figs. 5l and S5e).

We next hypothesized that rs7198799 mediated the activation of the NFATC2-ZFP90-BMP4 pathway in colorectal mucosa. To test this hypothesis, we detected the BMP4 expression in HCT116 cell lines with different genotype of rs7198799. We found that conversion of rs7198799 altered ZFP90 expression and downstream gene BMP4 expression level in HCT116 cells (Fig. 5m). We also found that knockdown of BMP4 partly blocked the rs7198799-dependent cell sphere formation alteration in HCT116 cells (Fig. 5n). Next, we genotyped SNP-rs7198799 and detected the mRNA level of NFATC2, ZFP90, and BMP4 with real-time PCR in normal colorectal mucosa in Cohort 1. The mRNA levels of NFATC2 in normal colorectal mucosa positively correlated with the levels of ZFP90 and negatively correlated with BMP4 level in CRC patients bearing rs7198799-CC genotype. However, this correlation became weaker in rs7198799-CT and TT genotype carriers (Fig. 5o). As expected, BMP4 expression is lower in normal colorectal mucosa with rs7198799-CC genotype compared with rs7198799-CT&TT genotype (Fig. S5f). Taken together, it is suggested that rs7198799 affects CRC tumorigenesis via the NFATC2-ZFP90-BMP4 pathway.

## Discussion

GWAS can efficiently identify disease susceptibility variants [29, 30]. However, the molecular bases and functional significance of the identified variants are often unclear. A major challenge, in the post-GWAS era, is to unravel the functional causal relationship between genetic variants and etiology. For example, the SNP rs9929218 at chromosome 16q22.1 has been identified as a high risk variant for CRC [14, 15]. However, its potential causal impact, gene target(s), and biological and clinical relevance remain to be defined in CRC.

Rs9929218, a CRC-associated tag SNP, resides within the second intron of the gene CDH1. We assume that it might be located in LD with unknown elements, controlling the expression of CDH1 or other vicinity genes. Our goal is to identify functional SNPs within the locus and their target genes, and to understand whether these target genes may contribute to CRC risk. eQTL analysis has chosen 12 genes around tag SNP rs9929218. Among the 12 genes, ZFP90 is the only transcript highly correlated with this SNP. Interestingly, the result is validated in eQTL analysis performed in normal colonic mucosa by Adria Closa et al. [18]. However, there is only a weak correlation between rs9929218 genotype and ZFP90 expression in GTEx database [31]. The difference in results may be due to different sample composition. To be specific, we and Adria Closa et al. performed eQTL analysis in normal mucosa, whereas colon specimens from GTEx study included muscularis. Next, 4C and 3C-qPCR data have illustrated a novel long-range interaction between the enhancer region (RL) containing a candidate SNP and the promoter of ZFP90. In support of our finding, GWAS studies suggest that potential causative variant may act on a distal gene [32]. For example, an 8q24 SNP is found to be situated within a transcriptional enhancer and can physically interact with and regulate the MYC proto-oncogene [33, 34]. Based on our eQTL, 4C, and 3C-qPCR data, we suggest that rs7198799 region may function as a ZFP90 distal enhancer. We have additionally characterized the regulatory landscape of causative variant to understand how the risk alleles affect gene function and carcinogenesis [9].

To date, CRC susceptible variants have rarely been functionally examined. In our current study, we show that the individuals bearing rs7198799-C risk alleles are prone to express high levels of ZFP90 in colorectal mucosa. Previous eQTL analysis in peripheral blood consistently suggested that ZFP90, rather than CDH1 or CDH3, is the most likely target of the16q22.1 genetic variation associated with increased CRC risk [17]. However, 16q22.1 region is considered a technically challenging region for causative variant identification since several highly correlated SNPs are closely located to the potential causal variant within 5 kb [17, 35]. Using 4C-seq and 3C-qPCR, we have successfully narrowed down the candidate causative variants to 18 SNPs. Next, the luciferase assay, with its ability to directly measure the functional effect of a variant on the enhancer activity, has reduced the candidates from 18 to 7 SNPs. The causative variant, rs7198799, has been finally identified through the single-site genome-editing approach, bioinformatics analysis, and an exhaustive screening strategy. In current study, the CRC-risk variant of rs7198799 was found to disrupt the binding of NFATC2. Interestingly, NFATC2 is a TF that can behave as an oncogene in colorectal tumorigenesis [36, 37]. Furthermore, it is demonstrated that the biological phenotypic difference among HCT116 cell lines with rs7198799-CC, TT, and parental CT largely depended on NFACT2-ZFP90 axis. Thus, we have established a novel complementary and comprehensive approach to identify a causative regulatory variant in a region with a highly complex LD structure. This strategy may be utilized for similar genetic studies in different types of human cancer.

ZFP90 is a zinc-finger protein containing KRAB box. Dysregulation of ZFP90 is associated with several diseases including obesity [25], cardiac dysfunction [38], and mental retardation [39]. As a potential target gene of 16q22.1 region, the function of ZFP90 in CRC is largely unknown. We have found that knockout of ZFP90 significantly decreased tumor formation capacity in CRC cells. Moreover, the oncogenic role of ZFP90 is validated in human xenograft models and an AOM-induced CRC mouse model with specific ZFP90 genetic deficiency in the host and in IEC. In addition, ZFP90 transcriptionally represses the expression of a tumor suppressor, BMP4, and mediates tumorigenesis. Thus, we have comprehensively dissected the oncogenic role of ZFP90, a previously unknown target gene of the causal SNP, in CRC initiation and progress. The genetic and biological importance of ZFP90 is further supported by our clinical studies. ZFP90 is consistently overexpressed in tumor tissues compared with adjacent normal mucosa, and is associated with poor CRC patient survival. Therefore, our work may help to explore novel therapy by targeting the interplay between the genetic risks of SNP and ZFP90 in patients with CRC.

Given the clinical, genetic, biochemical, and functional significance of 16q22.1 genetic variation and the defined functional target gene, ZFP90, we conclude that the risk SNP locus of rs7198799 and its associated pathways are crucial for colorectal carcinogenesis, and targeting this pathway may be pivotal in the prevention or treatment of CRC.

## Materials and methods

### Study population and clinical specimens

Two cohorts of patients with CRC were studied from Renji Hospital affiliated to Shanghai Jiao Tong University School of Medicine. Cohort 1 was enrolled between 2014 and 2017 in Renji Hospital. Cohort 2 was enrolled between 2009 and 2011 in Renji Hospital. All the CRC patients are Han Chinese. There were 239 snap frozen, colorectal cancerous tissues and paired adjacent normal colorectal mucosa in Cohort 1 and 90 formalin-fixed paraffin-embedded (FFPE) colorectal cancerous tissues and paired adjacent normal colorectal mucosa in Cohort 2. We extracted DNA and RNA from snap frozen tissues in Cohort 1 to detect gene transcripts and perform SNP genotyping. We used FFPE tissues in Cohort 2 to perform immunochemistry (IHC) and conduct survival analysis. In addition, clinical and pathological information was collected to perform related analyses.

### Cell lines

Human CRC cell lines, SW480, HCT116, and DLD1 were used in the study. All CRC cell lines were cultured according to ATCC culture methods. SW480 was cultured in L15 medium (GIBCO, Carlsbad, CA) supplemented with 10% fetal bovine serum (FBS) (GIBCO, Carlsbad, CA). The cell line was cultured at 37 °C without CO_2_. HCT116 was cultured in McCoy’s 5A medium (GIBCO, Carlsbad, CA) supplemented with 10% FBS. DLD1 was cultured in RPMI 1640 medium (GIBCO, Carlsbad, CA) supplemented with 10% FBS. These two cell lines were cultured at 37 °C with 5% CO_2_.

### Circular chromosome conformation capture (4C)

The 4C study was performed following an established protocol [40], with minor modifications. Cross-linking was performed by incubating cells in fresh medium supplemented with 2% formaldehyde for 10 min at room temperature. The reaction was quenched by addition of glycine to a final concentration of 0.125 M. Nuclei were harvested by lysis of the cells in ice-cold lysis buffer (10 mM Tris-HCl pH 8.0, 10 mM NaCl, 0.2% NP-40, and 1× complete protease inhibitor (Roche, Mannheim, Germany)) for 10 min on ice. The cross-linked DNA was digested with Hind III. Digested DNA was diluted with 6.125 ml 1.15× ligation buffer (NEB, UK) and ligated by T4 DNA ligase for 4 h at 16 °C. Proteinase K was added and DNA was incubated overnight at 65 °C to de-cross-link the samples. DNA was incubated for 30 min at 37 °C with RNase and purified. De-cross-linked DNA was digested overnight with Dpn II. Digested DNA was purified and ligated at low concentrations at 16 °C for 4 h. DNA was extracted by phenol–chloroform and ethanol precipitated with glycogen as a carrier. The resulting 4C templates were purified using the QIAquick PCR purification kit (Qiagen, Hilden, Germany). Restriction fragments containing ZFP90 TSS, RL (rs7198799), or RR (rs9929218) were used as bait. Bait sequence was listed in Table S5. 4C inverse PCR was performed using specific primers located on the bait fragment next to the restriction sites. Then, we pool all successful reactions from the same bait fragment and purify the DNA using QIAquick gel purification columns (Qiagen, Hilden, Germany) for paired-end sequencing according to the manufacturer Illumina.

### Chromatin immunoprecipitation combined with quantitative PCR (ChIP-qPCR) and sequencing (ChIP-seq)

ChIP analyses were performed on chromatin extracts from CRC cells according to manufacturer’s standard protocol (Merck Millipore, MA, USA). In brief, CRC cells were cross-linked with 1% formaldehyde, isolated with SDS lysis buffer. Subsequently, the chromatin was sonicated and immunoprecipitated with NFATC2 antibody (Abcam, MA, USA), ZFP90 antibody (SIGMA, MO, USA), and IgG (CST, MA, USA). DNA was extracted by Phenol–chloroform. We assessed enrichment of immunoprecipitated materials using PCR with gel electrophoresis and real-time PCR. DNA fragments were sequenced using Illumina. The detailed high-throughput sequencing was described in the data processing of high-throughput sequencing. ChIP-qPCR primers were listed in Table S5.

### AOM-induced CRC mice model

Eight-week-old male *Zfp90*^−/−^, *Zfp90*^fl/fl, Villin-cre/+^, and control mice were used in the experiments. Modeling and the following steps were conducted according to the published protocol [41]. Briefly, mice received intraperitoneal injection of AOM (10 mg/kg) in 10 weeks totally. Mice were sacrificed at week 8 after the last AOM injection. Colons were examined macroscopically, and fixed in formalin for subsequent HE staining and IHC experiments. Sample size was determined as common practice. No randomization was used. Investigators were not blinded during analysis.

### Statistical analyses

Statistical analyses were carried out using the program R (www.r-project.org). Data from at least three independent experiments performed in multiple replicates are presented as the means ± SD. Error bars in the scatterplots and the bar graphs represent SD. Data was examined to determine whether they were normally distributed with the one-sample Kolmogorov–Smirnov test. If the data was normally distributed, comparisons of measurement data between two groups were performed using independent sample *t* test. If the results showed significant difference, when the data presented skewed distribution, comparisons were performed by nonparametric test. Measurement data between two groups was compared using nonparametric Mann–Whitney test or Wilcoxon matched-pairs signed rank test. Spearman correlation analysis was performed to determine the correlation between two variables. Chi-square test was used to analyze the clinical variables. Survival data was analyzed using the standard Kaplan–Meier analysis and survival curves were compared using a log-rank test.

### Additional materials and methods

Further details on research design are available in the Supplementary Information and Table S6, including generation of SNP deletion/mutation cell lines, generation of Zfp90-knockout mice, and data processing of high-throughput sequencing.

## Supplementary information

Supplementary Information

Figure S1

Figure S2

Figure S3

Figure S4

Figure S5

Table S1

Table S2

Table S3

Table S4

Table S5

Table S6
