# Supplementary material for: A 16q22.1 variant confers susceptibility to colorectal cancer as a distal regulator of ZFP90
Source: Oncogene. 2019 Oct 22;39(6):1347–60. doi: 10.1038/s41388-019-1055-4 (PMC7002302; doi:10.1038/s41388-019-1055-4)
Supplement: Supplementary file 1 — Supplementary Information [file 41388_2019_1055_MOESM1_ESM.pdf]

Supplementary Information

Supplementary Materials and Methods

RNA Extraction and Real-time PCR

Total RNA was extracted from CRC cell lines and human colorectal tissues (colorectal cancerous tissues and paired adjacent normal colorectal mucosa) using AllPrep DNA/RNA Mini Kit (QIAGEN, Hilden, Germany) under manufacturer guidelines. 1 μg of total RNA was reverse transcribed using the PrimeScript RT Reagent Kit (Perfect Real Time; Takara, Japan) to detect relative mRNAs. Real-Time PCR was performed in triplicates on an Applied Biosystem7900 quantitative PCR system (Applied Biosystems, Foster City, CA) as described above. The Ct values obtained from different samples were compared using the $2^{-\Delta Ct}$ method. *GAPDH* served as the internal reference gene.

DNA Extraction and SNP Genotyping

Genomic DNA was extracted from four CRC cell lines and human colorectal tissues using AllPrep DNA/RNA Mini Kit (QIAGEN, Hilden, Germany). Sanger sequencing was used to detect genotypes including SNP-rs9929218 and SNP-rs7198799.

eQTL analysis

We tested for eQTL associations between genotypes of rs9929218-G/A, rs7198799-C/T and gene expression levels of twelve genes in 239 adjacent normal colorectal mucosa in Renji Cohort 1.We performed the expression quantitative trait loci analysis by fitting a linear regression model between the expression and the genotype data using the R

package ‘‘MatrixEQTL’’ version 2.1 (1). The p values were corrected for multiple testing using Bonferroni method.

Chromosome Conformation Capture combined with Quantitative PCR (3C-qPCR)

3C-qPCR was performed according to the method as Hagege et al. described(2). Briefly, 10^7 cells were fixed with 1% formaldehyde and fractionated for nuclear fraction. Nuclear lysates were digested with 400 U Hind III overnight at 37°C. Digested chromatin fragments were incubated at 16°C overnight in ligation buffer (750 mL of 10% Triton X-100, 750 mL of 10 3 NEB ligation buffer, 75 mL of 10 mg/ml BSA, 5,925 mL of distilled water and 4,000 U T4 DNA ligase (M0202S, New England Biolabs)). DNA was extracted with phenol-chloroform and subjected to PCR and qPCR amplifications using primers listed in **Table S5**.

Enhancer Scanning

RL was evenly tiled into five segments (S1 - S5) and artificially synthesized each into the cloning site of the pGL3-LRF and pGL3-LRR. Two recombinant vectors, pGL3-LRF and pGL3-LRR, are used to clone desired tiling clones by recombination. Based on a recent report(3), at day 1, we plated HCT116 and DLD1 cells in 100 µl of cell culture medium in a 96-well plate and incubate them for 24 hours. At day 2, plasmids containing tiling clones in both orientations were individually transfected in HCT116 and DLD1 cells using FuGene HD (Promega, Wisconsin, U.S.A.). To ensure data quality, we included a positive control (pGL3-control vector), two negative controls (pGL3-LRF and pGL3-LRR), and an internal control (pRL-CMV Renilla). At day 3, we used Dual-Glo Luciferase Assay System (Promega, Wisconsin, U.S.A.) to measure Renilla and firefly

luciferase activity 24 hours after transfection. The luciferase activity was measured using a FLUOstar Omega (BMG Labtech, Ortenberg, Germany) in eight replicates.

Generation of SNP Deletion/Mutation Cell Lines

△S3-S5 HCT116: For generating a fragment deleted cell line, two sgRNAs were designed to target 4632 bp between positions 68781377-68786008 of Chromosome 16 (hg 38). For each targeting site, candidate guide RNAs were designed by the CRISPR design tool (http://crispr.mit.edu/). Guide RNAs were screened for on-target activity use MSDase assay.

To minimize random integrations, we employed a circular donor vector. The targeting vector containing PuroDeltatk cassette and 2 homology arms of left (800 bp) and right (800 bp) each was used as a template to repair the DSBs generated by Cas9/sgRNA. 4632 bp of sequence encompassing SNPs was replaced by PuroDeltatk cassette, resulting in 4632 bp deletion. sgRNA6, sgRNA11 and targeting vectors were electroporated into HCT116 cell line. After drug resistance selection, we extracted the DNA of the mixed clones and identified positive ones by PCR and sequencing. Then, 85 clones were obtained by limiting dilution analysis of positive mixed clone. Finally, 6 homozygous clones were obtained by further genotyping and PCR product sequencing.

△RR HCT116 and △S2 HCT116 were constructed in a similar way to △S3-S5 HCT116.

SNP-rs7198799/TT HCT116: For generating a point mutation knock in cell line, sgRNAs were designed to target upstream or downstream regions of the position 68784487 of Chromosome 16 (hg 38). For the targeting site, candidate guide RNAs were

designed by the CRISPR design tool (http://crispr.mit.edu/). Guide RNAs were screened for on-target activity using MSDase assay.

To minimize random integrations, we employed a circular donor vector. The targeting vector, containing PuroDeltatk cassette and 2 homology arms of left (814 bp) introducing a point mutation with a single base substitution (chr16: 68784487 C>T) and right (1000 bp) each, was used as a template to repair the DSBs generated by Cas9/sgRNA. sgRNA9 and targeting vectors were electroporated into HCT116 cell line. After drug resistance selection, we extracted the DNA of the mixed clones and identified positive ones by PCR and sequencing. Then, 123 clones were obtained by limiting dilution analysis of positive mixed clone. Finally, 2 homozygous clones were obtained by further genotyping and PCR product sequencing.

SNP-rs7198799/CC HCT116 and SNP-rs7199991/GG HCT116 were constructed in a similar way to SNP-rs7198799/TT HCT116.

Western Blot and Chemical Reagents

Western blot was performed using standard protocols. Cell extracts were collected and quantified using BCA Protein Assay Kit (Thermo Scientific, Rockford, U.S.A.). 40 μg of protein was electrophoresed through 10% SDS polyacrylamide gels and were then transferred to PVDF membranes (Bio-Rad, Hercules, CA). The membranes were blocked in 5% BSA for one hour and then incubated with primary antibodies at 4°C overnight. Secondary antibodies were labeled with HRP (KangChen, China) and the signals were detected using Pierce ECL Western Blotting Substrate (Thermo Scientific, Rockford, U.S.A.) by ChemiDoc Imaging System (BIO-RAD, U.S.A.). A GAPDH antibody was

used as a control for whole-cell lysates. The information on all antibodies is listed in **Table S6**.

Electrophoretic Mobility Shift Assays (EMSA)

Oligonucleotides were synthesized for the region surrounding the rs7198799 SNP in the second intron of CDH1 to create 41-bp probes.

The sequence for the probe with NFATC2 consensus oligonucleotide were as follows: (F) 5'-CGGAGGAAAACTGTTTCATACAGAAGGCGTG-3' and (R) 5’-CACGCCTTCTGTATGAAACAGTTTTCCTCCG-3’.

The sequences for the probe with the C genotype were as follows: (F) 5'-TGTGTGGGTCATCCTCCTTTCCACCGCCCACATGGACCCCT-3' and (R) 5'-AGGGGTCCATGTGGGCGGTGGAAAGGAGGATGACCCACACA-3'.

The sequences for the probe with the T genotype were as follows (F)5’-TGTGTGGGTCATCCTCCTTTTCACCGCCCACATGGACCCCT-3' and (R) 5'-AGGGGTCCATGTGGGCGGTGAAAAGGAGGATGACCCACACA-3'.

The sequence for the unrelated probe were as follows: (F) 5'-GAGATTTTGGTGCACCCATGATTGGAGCAGTGTACACTGTA-3' and (R) 5'-TACAGTGTACACTGCTCCAATCATGGGTGCACCAAAATCTC-3'.
Oligonucleotides were biotinylated at the 3′ end. Nuclear extracts were prepared from HCT116 cells using the CelLytic NuCLEAR Extraction Kit (SIGMA, Missouri, U.S.A.). EMSA was conducted using the LightShift Chemiluminescent EMSA Kit (Thermo Scientific, Rockford, U.S.A.) according to the manufacturer’s instructions. Briefly,

negative control was set without nuclear protein. Double-stranded biotinylated NFATC2-consensus oligonucleotides were incubated for 20 min with nuclear protein in the presence of no competitor or 50-fold molar excess of unrelated, NFATC2-consensus, C allele or T allele double stranded competitor oligonucleotides.

The resulting reactions were resolved in a 4% nondenaturing PAGE gel and run at 100V for 50min, transferred onto nylon membrane (380 mA, 60 min) in the ice box. DNA was cross-linked to the membrane using the UV light in the laminar flow bench. Then the complexes were detected using streptavidin-HRP conjugate and a chemiluminescent substrate.

Tumor Sphere Assay

For tumor sphere assay, 500 *ZFP90*-KO and WT colorectal cancer cells including HCT116 and DLD1 were plated on ultralow attachment 96-well plates (Corning, New York, U.S.A.) and grown in DMEM/F12 medium (serum free) supplemented with 1% BSA, 1 x B27 (Gibco), 20 ng/ml EGF (Gibco), 20 ng/ml bFGF (Gibco) and insulin (Gibco). Tumor spheres were counted and photographed at day 7 under microscope. To explore the role of *NFATC2* and *BMP4* in *ZFP90*-guided tumor sphere formation ability, *NFATC2* and *BMP4* siRNA transfection were conducted as described above.

RNA Interference

Oligonucleotide Transfection siRNAs of *ZFP90*, *BMP4* and *NFATC2* were purchased from Genepharma (Shanghai, China). Oligonucleotide transfection was performed using the DharmaFECT 1 siRNA transfection reagent, while nonspecific siRNAs were used as negative controls.

Plasmids Constructions and Transfection

The recombinant vector encoding human *ZFP90* (Gene ID: 146198, 636 aa in ZFP90 protein) was cloned into the pcDNA3.1 framework. Cells were seeded on culture plate 24 hours before transfection. Plasmids were transfected using FuGene HD according to the manufacturer's instruction.

Generation of *ZFP90*-Knockout Cell Lines

To create *ZFP90*-KO alleles in HCT116, sgRNAs were designed to target exon 3 of *ZFP90* as described previously. The sgRNAs sequences were cloned into the pSpCas9(BB)-2A-Puro (Px459) plasmid. HCT116 cells were transfected by electroporation. 24 hours after electroporation, the culture medium was replaced. Cells were cultured in medium with 4 μg/ml puromycin for 48 hours for clone selection. Consistent clones were picked and transferred to a 96-well plate for further screening. Finally, extracted DNAs were used for Sanger sequencing to verify the on-target and off-target editing(4). DLD1 *ZFP90*-KO cell line was constructed according to the method as HCT116 *ZFP90*-KO cell line.

GSEA

Single-sample gene set enrichment analysis (ssGSEA) was used to assess gene set activation scores in gene expression profiling data. ssGSEA calculates a sample level gene set score by comparing the distribution of gene expression ranks inside and outside the gene set. The ssGSEA score was calculated by Gene Set Variation Analysis (GSVA) R package(5). Statistical tests were two-tailed and a p value of less than 0.05 was considered statistically significant.

Xenograft Experiments

4-week-old male BALB/c nude mice were housed in laminar flow cabinets under specific pathogen-free conditions.

To explore the role of ZFP90 on tumor growth, 5 x 10^6 ZFP90-KO and WT HCT116 cells were injected subcutaneously into the right axilla of each mouse to establish the CRC xenograft model.

Three weeks after tumor inoculation, all mice were sacrificed and tumors were collected and weighed. The tumor volume and weight were presented as means ± SD.

The length and width of the tumors (in millimeters) were measured every three days with calipers. Tumor volume was calculated using the formula $(A \times B^2)/2$, where A and B are the long and short dimensions, respectively.

4-week-old male NOD/Shi-scid/IL-2Rγnull (NSG) mice were housed in laminar flow cabinets under specific pathogen-free conditions. To explore the role of ZFP90 on tumor formation potential, we used 4-week-old male NOD/Shi-scid/IL-2Rγnull (NSG) mice. 10^2, 10^3, 10^4, 10^5, and 10^6 ZFP90-knockout and WT HCT116 cells were subcutaneously inoculated into the NSG mice. Tumor incidence was evaluated for 4 weeks after inoculation.

Generation of *Zfp90*-Knockout Mice

The heterozygotes of *Zfp90* global knockout (*Zfp90*$^{+/-}$) mice were generated by CRISPR/Cas9-mediated genome editing via nonhomologous end joining. Cas9 mRNA

and sgRNA were injected into C57BL/6J ES cells to target exon 2 and 3 of the *Zfp90* gene.

For the generation of the *Zfp90*$^{fl/+,Villin-cre/+}$ mice, exon 4 and 3'UTR were flanked by loxP sites using C57BL/6J ES cells with a 2kb fragment upstream and downstream as the 5' and 3' arms. Then, the *Zfp90*$^{fl/+}$mice were crossed to C57BL/6J Villin-cre mice to generate *Zfp90*$^{fl/+,Villin-cre/+}$ mice.

Homozygous knockout and control mice were generated from *Zfp90*$^{+/-}$ and *Zfp90*$^{fl/+,Villin-cre/+}$ mice.

Mice were genotyped via PCR and Sanger sequencing using genomic DNA obtained from mice tails.

Mouse experiments were conducted in accordance with the National Institutes of Health Guidelines for the Care and Use of Laboratory Animals. The study procedures were approved by the Institutional Animal Care and Use Committee of Renji Hospital, School of Medicine, Shanghai Jiao Tong University.

Immunohistochemistry (IHC)

For immunohistochemistry, 4-µm-thick paraffin colon tissue slides were subjected to heat-induced antigen retrieval. After blocking with 10% sheep serum in PBS, slides were incubated with ZFP90, BMP4, Ki67 antibody in a humidified chamber at 4°C overnight. Then, the slides were incubated with secondary antibody at room temperature for 1 hour and stained with DAB substrate solution to reveal the staining.

Tumor Colonoids

Colon tumor colonoids were isolated from both Zfp90$^{-/-}$ and WT C57BL/6J mice according to previously reported protocols(6,7). The mouse tumor colonoids culture medium contained basal medium (Advanced Dulbecco's modified Eagle medium/F12 [Gibco]), supplemented with penicillin/streptomycin (Gibco), 10 mmol/L HEPES (Gibco), 2 mmol/L GlutaMAX (Gibco), 1 x B27 (Gibco), and 1 x N2 (Gibco)] and growth factors (50 ng/mL EGF [Gibco], and Noggin (PeproTech)). Tumor colonoids diameters were measured every 24 hours under microscope using software ruler to observe growth ability.

Data Processing of High-Throughput Sequencing

4C data processing: A reduced genome was generated by extracting the sequences flanking the Hind III restriction sites (30 bp on each strand from the Hind III restriction sites to downstream) using the hg19 reference human genome to improve the mapability of our 4C data. The reads from each library were parsed based on the bait-specific primer sequence and mapped to the reduced hg19 genome using bowtie2 with the default parameters. A Bioconductor package 'r3Cseq' (8) with 2.5 kb sliding window was used to determine significant interactions and calculate interaction difference. The raw data are accessible through GEO Series accession number GSE121623. (reviewer access token: qpepkikyfdgffil)

ChIP-seq data processing: The FASTQ files were aligned to hg19 using Bowtie2. Enriched regions were determined by the MACS1.4 program with default setting(9). ChIP-seq density heatmaps and histograms were generated using ngs.plot(10). The raw

data is accessible through GEO Series accession number GSE121622. (reviewer access token: qpepkikyfdgffil)

RNA-seq data processing: Samples were sequenced in an Illumina HiSeq 3000 for 2 × 150-bp paired-end sequencing. Reads were mapped to the human genome (hg19) using TopHat v2.0.117 (http://tophat.cbcb.umd.edu) with the following default options with a TopHat transcript index built from Ensembl_GRCh37(11). Transcript expression was estimated with an improved version of Cuffdiff (http://cufflinks.cbcb.umd.edu). Cuffdiff was run with the default options against the UCSC iGenomes GTF file from Illumina (http://cufflinks.cbcb.umd.edu/igenomes.html). The workflow used to analyze the data was described in detail in Trapnell et al(12) . To identify a gene or transcript as differential expression, Cuffdiff2 tests the observed log-fold-change in its expression against the null hypothesis of no change (i.e., the true log-fold-change is zero). Clustering of gene expression profiles was achieved with the csDendro function from CummeRbund (http://compbio.mit.edu/cummeRbund/). The RNA sequence data has been deposited in NCBIs Gene Expression Omnibus (GEO, http://www.ncbi.nlm.nih.gov/geo/) and is accessible through GEO Series accession number GSE121621. (reviewer access token: qpepkikyfdgffil)

References

1. Shabalin, A.A. (2012) Matrix eQTL: ultra fast eQTL analysis via large matrix operations. *Bioinformatics*, **28**, 1353-1358.
2. Hagege, H., Klous, P., Braem, C., Splinter, E., Dekker, J., Cathala, G., de Laat, W. and Forne, T. (2007) Quantitative analysis of chromosome conformation capture assays (3C-qPCR). *Nature protocols*, **2**, 1722-1733.
3. Buckley, M., Gjyshi, A., Mendoza-Fandino, G., Baskin, R., Carvalho, R.S., Carvalho, M.A., Woods, N.T. and Monteiro, A.N. (2016) Enhancer scanning to locate regulatory regions in genomic loci. *Nat Protoc*, **11**, 46-60.
4. Shalem, O., Sanjana, N.E., Hartenian, E., Shi, X., Scott, D.A., Mikkelson, T., Heckl, D., Ebert, B.L., Root, D.E., Doench, J.G. *et al.* (2014) Genome-scale CRISPR-Cas9 knockout screening in human cells. *Science*, **343**, 84-87.
5. Hanzelmann, S., Castelo, R. and Guinney, J. (2013) GSVA: gene set variation analysis for microarray and RNA-seq data. *BMC bioinformatics*, **14**, 7.
6. Xue, X. and Shah, Y.M. (2013) In vitro organoid culture of primary mouse colon tumors. *J Vis Exp*, e50210.
7. Fujii, M., Matano, M., Nanki, K. and Sato, T. (2015) Efficient genetic engineering of human intestinal organoids using electroporation. *Nature protocols*, **10**, 1474-1485.
8. Thongjuea, S., Stadhouders, R., Grosveld, F.G., Soler, E. and Lenhard, B. (2013) r3Cseq: an R/Bioconductor package for the discovery of long-range genomic interactions from chromosome conformation capture and next-generation sequencing data. *Nucleic Acids Res*, **41**, e132.
9. Zhang, Y., Liu, T., Meyer, C.A., Eeckhoute, J., Johnson, D.S., Bernstein, B.E., Nusbaum, C., Myers, R.M., Brown, M., Li, W. *et al.* (2008) Model-based analysis of ChIP-Seq (MACS). *Genome Biol*, **9**, R137.
10. Shen, L., Shao, N., Liu, X. and Nestler, E. (2014) ngs.plot: Quick mining and visualization of next-generation sequencing data by integrating genomic databases. *BMC Genomics*, **15**, 284.
11. Trapnell, C., Pachter, L. and Salzberg, S.L. (2009) TopHat: discovering splice junctions with RNA-Seq. *Bioinformatics*, **25**, 1105-1111.
12. Trapnell, C., Roberts, A., Goff, L., Pertea, G., Kim, D., Kelley, D.R., Pimentel, H., Salzberg, S.L., Rinn, J.L. and Pachter, L. (2012) Differential gene and transcript expression analysis of RNA-seq experiments with TopHat and Cufflinks. *Nat Protoc*, **7**, 562-578.

Supplementary Figure Legends

Fig. S1 CRC-Risk Haplotype at 16q22.1 Interacts with ZFP90 in Colon epithelium.

a Locus zoom plot of 16q22.1 genetic association is shown in CRC. SNPs and CRC risk were demonstrated in six case–control studies of European population (According to the result of fine-mapping at 16q22.1 locus by Carvajal-Carmona et al., 2011). The x-axis indicates the SNP location based on the human genome build 37 (hg 19). The left y-axis indicates the -log10 of the association P-value by fine-mapping. The right y-axis indicates recombination rate. Each variant identified in 1000 Genomes Project sequencing is represented by a dot, with color representing linkage disequilibrium (LD) with the tag SNP rs9929218. **b** Sanger sequencing of three different genotypes of SNP-rs9929218-G/A in human colorectal mucosa. **c** 4C identified chromatin interactions between *ZFP90* region and SNP region (containing RR and RL) in SW480 cells. *ZFP90* promoter served as anchor. **d** 4C identified chromatin interactions between RR and the *ZFP90* region in SW480 and HCT116 cells. RR served as anchor. **e** 3C-qPCR was performed to determine the relative interaction frequencies between RR (containing SNP-rs9929218) and *ZFP90* promoter region, comparing the relative abundance of ligation products formed between the fragment mapping to RR and each of the target fragments in *ZFP90* promoter region. Results are normalized to the relative abundance of control region. n = 3 with triplicates, non-paired t-test, SW480 vs HCT116. **f** 3C-qPCR was performed to determine the relative interaction frequencies between *ZFP90* promoter region and SNP region (containing RR and RL), comparing the relative abundance of ligation products formed between the fragment mapping to *ZFP90* promoter region and

each of the target fragments in RL and RR region. Results are normalized to the relative abundance of control region. n = 3 with triplicates, non-paired t-test, SW480 vs HCT116. **g** Real-time PCR was performed to determine expression changes in mRNA levels of ZFP90 with deletion of region right (△RR) containing SNP-rs9929218 in HCT116.

Fig. S2 SNP-rs7198799 is a Causal Variant Constituting an ZFP90 Distal Enhancer.

a Enhancer RNA was measured in different types of human cells of epithelial origin from FANTOM5 database. The detection area is chr16: 68784311-68784444 (hg 38). **b** RL was evenly divided into 5 segments for enhancer activity detection with luciferase reporter assay. 7 SNPs in S5 were amplified. **c, d, e** Luciferase reporter assay was performed to detect the enhancer activity of the risk and non-risk haplotypes in different orientations in DLD1 (C, D) and HCT116 (E) cells. n = 3 with eight replicates, non-paired two-tailed t-test, RH (risk haplotype) vs NR (non-risk haplotype). **f** Scheme of generation of HCT116 cell lines with 4632-bp deletion (△S3-S5, containing SNP-rs7198799). **g** Real-time PCR was performed to determine expression changes in mRNA levels of *ZFP90* and *CDH1* with deletion of rs7198799 regulatory region (△S3-S5). n = 3 with triplicates, non-paired two-tailed t-test, WT vs △S3-S5. **h** Western blot was performed to determine expression changes in protein levels of *ZFP90* and *CDH1* with deletion of rs7198799 regulatory region (△S3-S5). **i** Western blot was performed to determine expression change in protein levels of *ZFP90* and *CDH1* with deletion of S2 (△S2). **j** Luciferase reporter assay was performed to detect the enhancer activity. Each SNP was mutated from the risk allele to the non-risk allele in the

background of S5 with risk haplotype in DLD1 cells. n = 3 with eight replicates, non-paired two-tailed t-test, S5-RH with rs7198799 non-risk allele vs S5-RH with rs7198799 risk allele. **k** Scheme of generation of HCT116 cell lines with SNP-rs7198799 mutation (CT > TT/CC). **l** Real-time PCR was performed to determine the expression change in mRNA levels of *ZFP90* and *CDH1* with SNP-rs7199991 mutation (TG > GG). n = 3 with triplicates, non-paired two-tailed t-test, SNP-rs7199991_TG vs SNP-rs7199991_GG. **m** Western blot was performed to determine expression change in protein levels of ZFP90 and CDH1 with SNP-rs7199991 mutation (TG > GG). **n** Sanger sequencing of three different genotypes of SNP-rs7198799-C/T in human colorectal mucosa.

Fig. S3 Differential Activity of rs7198799 Is Mediated by NFATC2.

a The motif changes by rs7198799 was accessed by SNPInspector. **b** Workflow of causal variant identification in CRC susceptibility locus 16q22.1.

Fig. S4 ZFP90 Affects Colorectal Tumorigenesis *in vitro* and *in vivo.*

a *ZFP90* expression in CRC tissues and adjacent normal colorectal tissues in TCGA database. Non-parametric Mann–Whitney test, adjacent tissue vs cancerous tissue. **b** Representative immunohistochemical staining of ZFP90 proteins in CRC tissues and paired normal colorectal mucosa. n = 90, Cohort 2. **c** Statistical analysis of immunohistochemical IRS scores of ZFP90 proteins in CRC. Wilcoxon matched-pairs

signed rank test, adjacent colorectal tissue vs cancerous tissue. **d** GSEA was used to identify the differential gene profiles between ZFP90-high CRC tumors and ZFP90-low CRC tumors in TCGA datasets. NES, normalized enrichment score. **e** Comparing different histological grades and AJCC stage between CRC patients with different ZFP90 expression in CRC tissue. n = 90, Cohort 2. The heatmap illustrates the association of different clinical characters with ZFP90 expression level in CRC tissue. Statistical significance was performed by the Chi-square test. **f** Survival analysis was performed between patients with low and high protein level of ZFP90 in CRC tissues. Cohort 2. Log-rank test. **g** Overall Survival (OS) was compared between CRC patients with low and high expression of *ZFP90* in GSE17536, Log-rank test. **h** Data of tumors is shown in NSG model after inoculation with 10^2, 10^3, 10^4, 10^5, 10^6 HCT116 cells (WT or *ZFP90*-KO). N = 8/group. Tumor incidence was concluded in the table. Rank-sum test. **i** Representative data of tumors in nude mice bearing HCT116 cells (WT or *ZFP90*-KO). **j** Scheme of establishment of global *Zfp90*-KO mice by CRISPR/Cas9 system. **k** Scheme for the experimental course of AOM-induced colon carcinogenesis. **l** Average tumor volume for WT mice and $Zfp90^{-/-}$ mice. n = 6, Non-parametric two-tailed Mann–Whitney test, WT vs $Zfp90^{-/-}$. **m** Average tumor volume for Control mice and $Zfp90^{fl/fl,Villin\text{-}cre/+}$ mice. n = 5, Non-parametric two-tailed Mann–Whitney test, Control vs $Zfp90^{fl/fl,Villin\text{-}cre/+}$. **n** Scheme of establishment of intestinal *Zfp90*-KO ($Zfp90^{fl/fl, Villin\ cre/+}$) mice by CRISPR/Cas9 system. **o** Survival curve of WT mice compared with $Zfp90^{-/-}$ mice after a 10-week cycle of 10 mg/kg AOM treatment. n = 5, Log-rank test.

Fig. S5 ZFP90 Targets BMP4 to Control Carcinogenesis.

a Heatmap of ZFP90 global genomic binding at the target sites in HCT116 cells. **b** Functional pathways of ZFP90 binding cis-regulatory regions were predicted by GREAT algorithm. **c** Real-time PCR was performed to determine the mRNA levels of *BMP4*, *FXYD4*, *GATA2*, *CASP10*, *SERPING1* and *PED4D* in HCT116 cells. HCT116 cells were transfected with *ZFP90* siRNA or N.C. siRNA. n = 3 with replicates, non-paired two-tailed t-test, N.C. siRNA vs *ZFP90* siRNA. **d** Real-time PCR of the ChIP samples was performed to determine ZFP90 binding efficiency to *BMP4*, *GATA2*, *SERPING1* and *PDE4D* promoter. n = 3 with replicates, non-paired two-tailed t-test, IgG vs ZFP90 Ab. **e** Western blot was performed to determine the protein levels of Zfp90 and Bmp4 in WT mice and *Zfp90*$^{-/-}$ mice, respectively. **f** Real-time PCR was performed to determine the mRNA level of *BMP4* in human normal adjacent colorectal tissue (SNP-rs7198799_CC or SNP-rs7198799_CT & TT). Non-parametric two-tailed Mann–Whitney test, CC vs CT & TT.
