# Supplementary figures and images for: A 16q22.1 variant confers susceptibility to colorectal cancer as a distal regulator of ZFP90
Source: Oncogene. 2019 Oct 22;39(6):1347–60. doi: 10.1038/s41388-019-1055-4 (PMC7002302; doi:10.1038/s41388-019-1055-4)

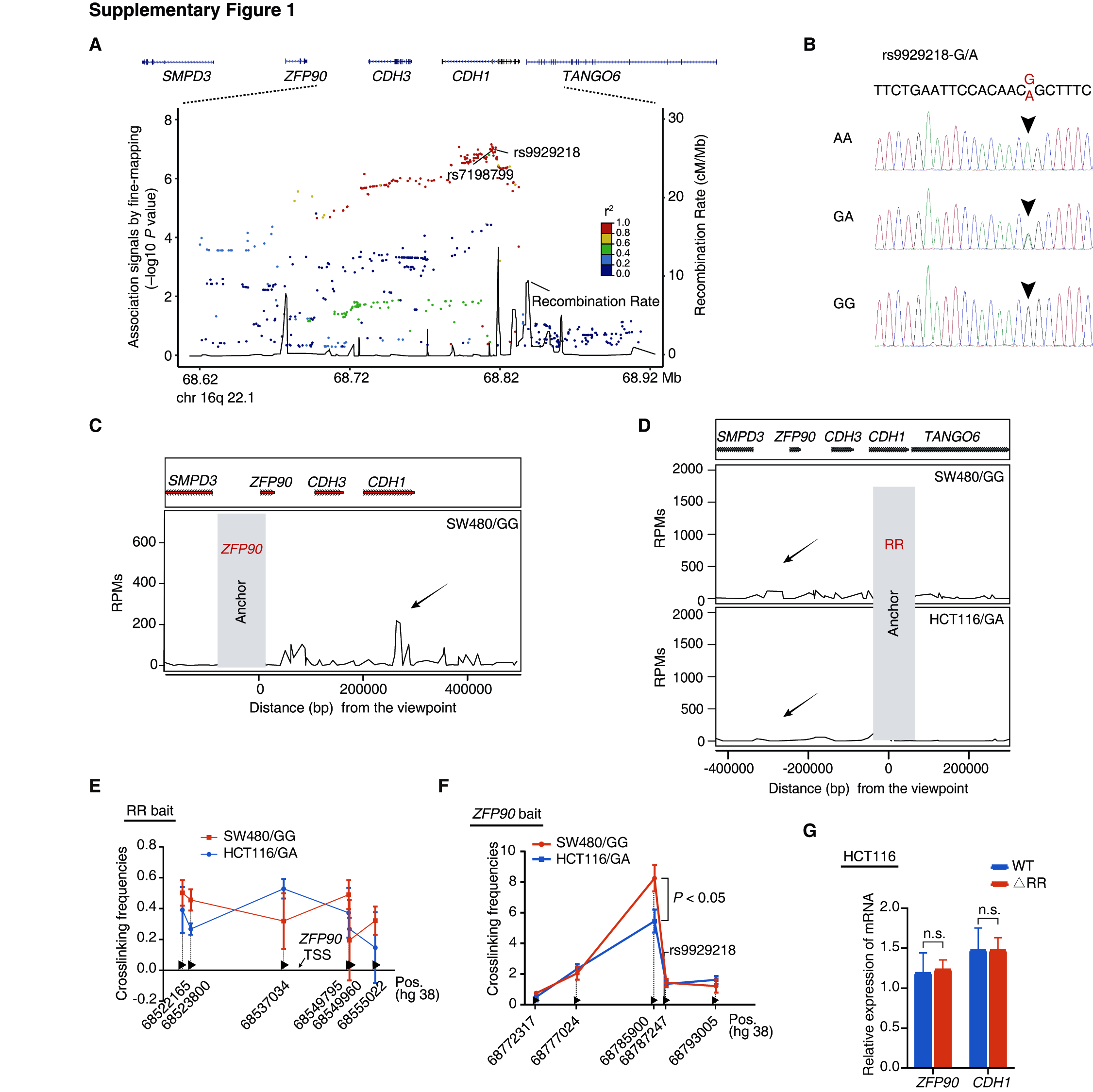

Supplement: Supplementary file 2 — Figure S1 [file 41388_2019_1055_MOESM2_ESM.tif]

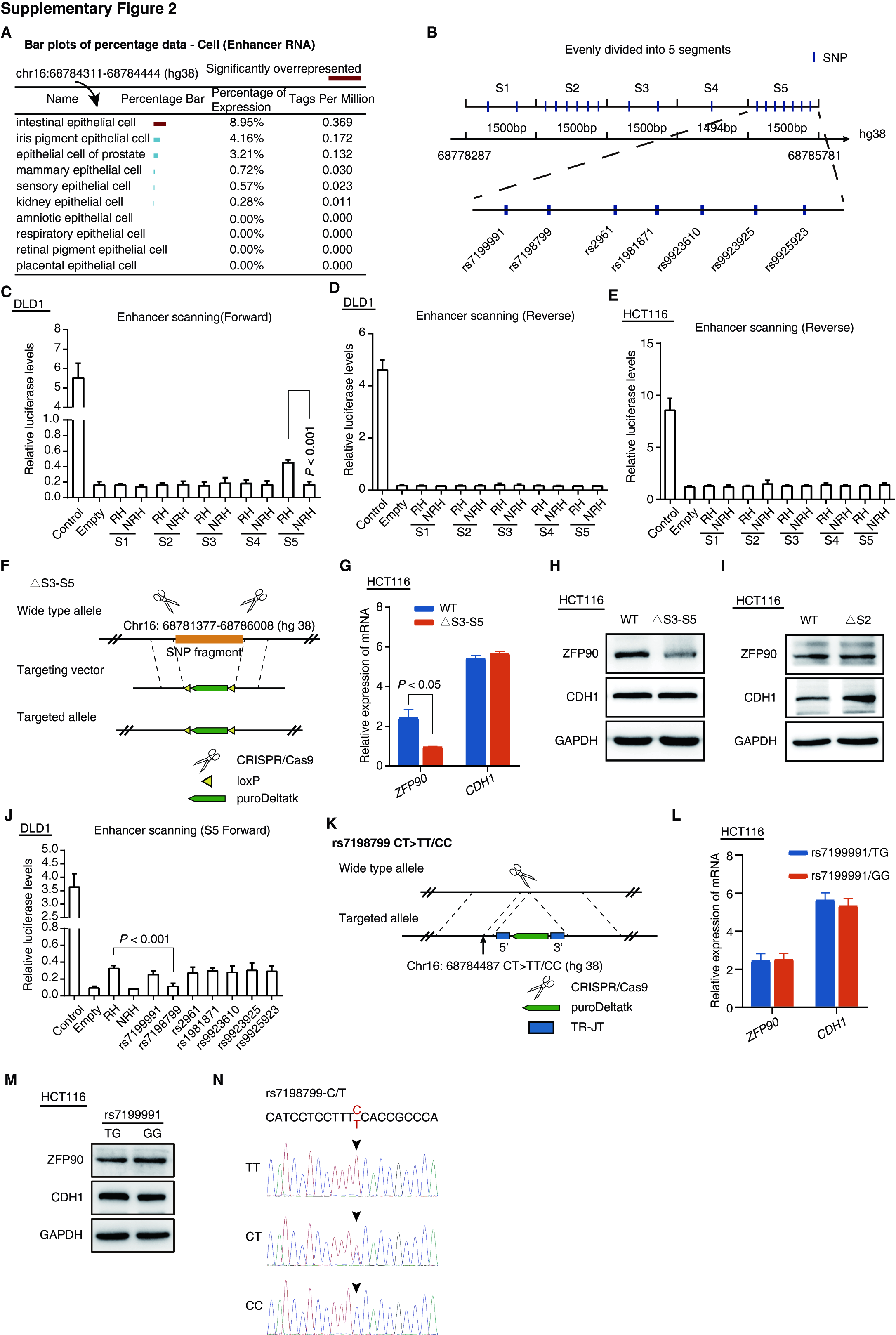

Supplement: Supplementary file 3 — Figure S2 [file 41388_2019_1055_MOESM3_ESM.tif]

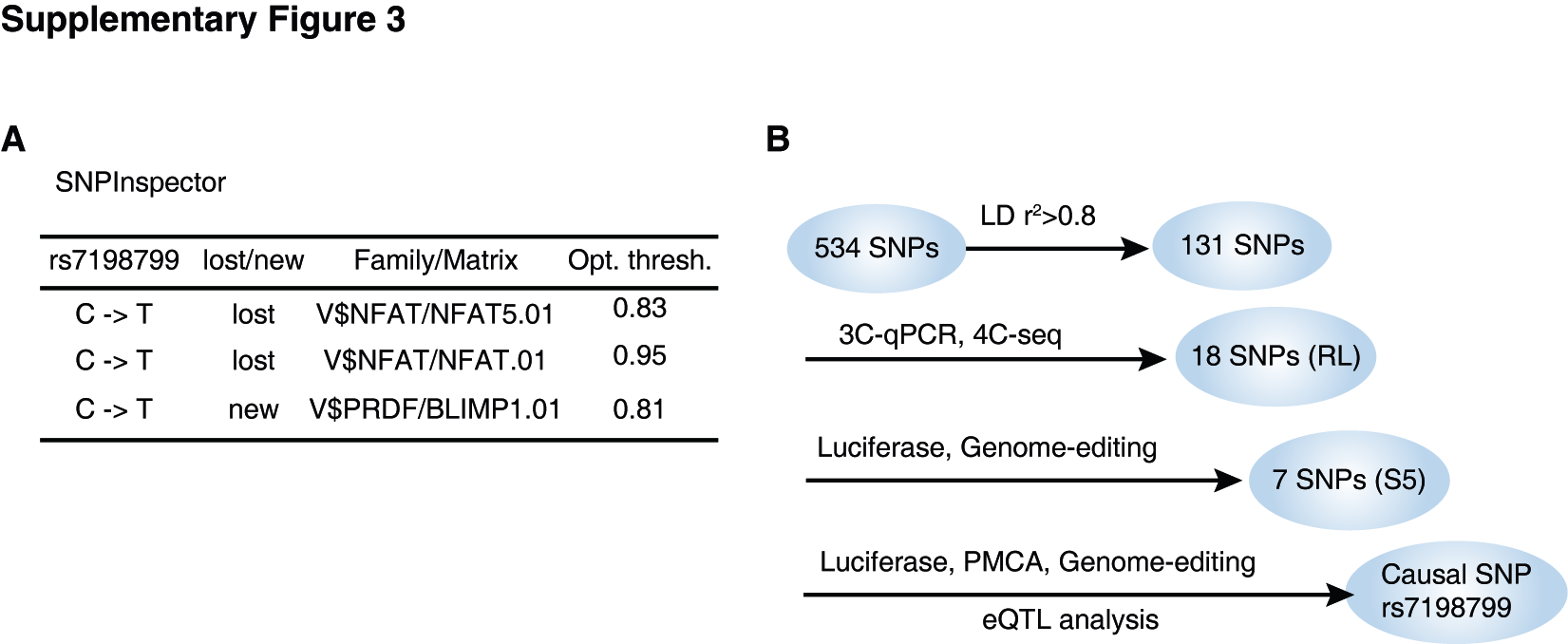

Supplement: Supplementary file 4 — Figure S3 [file 41388_2019_1055_MOESM4_ESM.tif]

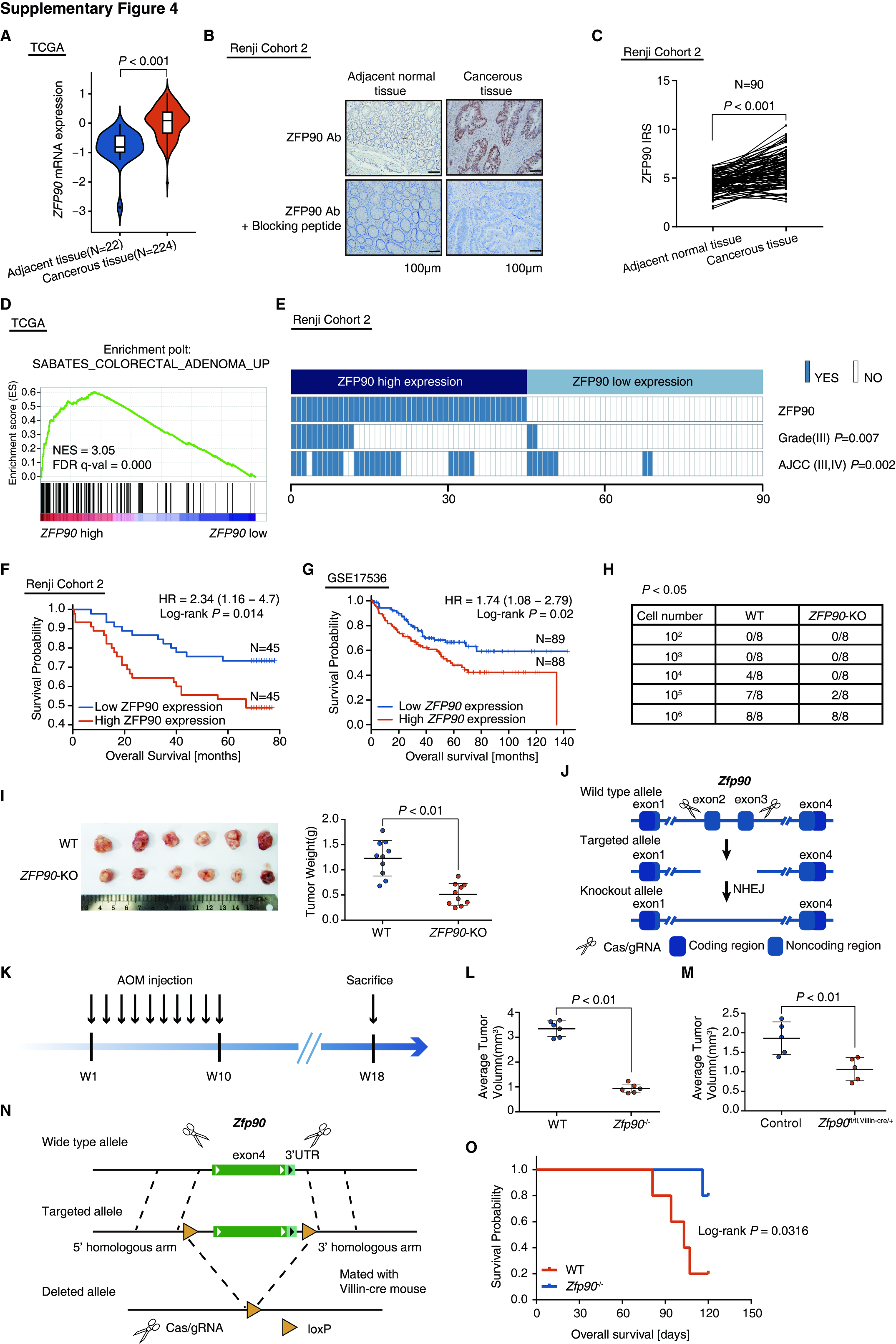

Supplement: Supplementary file 5 — Figure S4 [file 41388_2019_1055_MOESM5_ESM.tif]

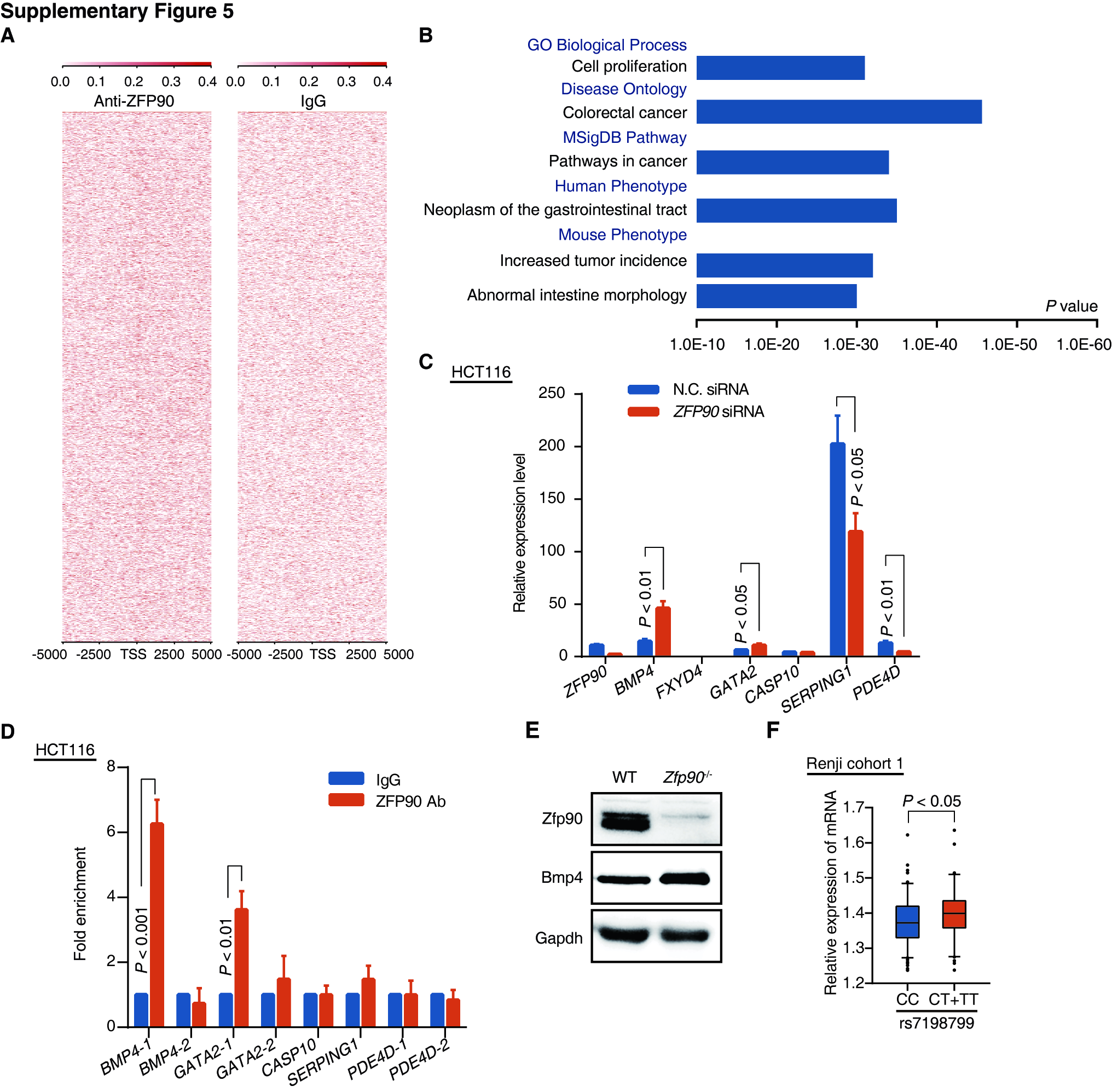

Supplement: Supplementary file 6 — Figure S5 [file 41388_2019_1055_MOESM6_ESM.tif]
