# Supplementary material for: A 16q22.1 variant confers susceptibility to colorectal cancer as a distal regulator of ZFP90
Source: Oncogene. 2019 Oct 22;39(6):1347–60. doi: 10.1038/s41388-019-1055-4 (PMC7002302; doi:10.1038/s41388-019-1055-4)
Supplement: Supplementary file 7 — Table S1 [file 41388_2019_1055_MOESM7_ESM.pdf]

Table S1: Clinical information in Cohort 1 (snap frozen tissues)

Number	Gender	Age	Location	TNM Stage
1	Male	50	ascending colon	T3N0M0
2	Female	52	sigmoid colon	T4N0M0
3	Female	73	sigmoid colon	T3N0M0
4	Male	31	rectum	T2N0M0
5	Male	65	ileocecal junction	T3N0M0
6	Female	59	descending colon	T3N0M0
7	Male	86	descending colon	T4N1M0
8	Female	74	rectum	T3N0M0
9	Male	80	transverse colon	T2N0M0
10	Female	72	rectum	T3N0M0
11	Male	84	sigmoid colon	T3N0M0
12	Male	60	splenic flexure	T4N0M1
13	Male	66	descending colon	T4N0M0
14	Male	72	rectum	T4N1M0
15	Female	73	descending colon	T3N0M0
16	Male	60	descending colon	T4N0M0
17	Female	65	splenic flexure	T3N0M0
18	Female	65	rectum	T2N0M0
19	Male	67	descending colon	T3N0M0
20	Male	61	rectum	T3N0M0
21	Male	66	rectum	T2N0M0
22	Female	68	ascending colon	T3N0M0
23	Male	38	rectum	T3N1M0
24	Female	63	ascending colon	T1N0M0
25	Male	79	rectum	T2N1M0
26	Female	44	ileocecal junction	T4N1M0
27	Female	51	rectum	T2N0M0
28	Female	84	ascending colon	T4N0M0
29	Male	74	hepatic flexure	T3N1M0
30	Female	47	rectum	T4N1M0
31	Female	78	rectum	T2N0M0
32	Male	50	rectum	T4N2M0
33	Male	61	descending colon	T4N1M0
34	Male	60	rectum	T4N1M1
35	Male	69	rectum	T4N1M0
36	Male	54	splenic flexure	T4N0M0
37	Male	86	splenic flexure	T3N1M0
38	Male	50	rectum	T4N2M0
39	Female	62	rectum	T3N0M0
40	Female	69	ascending colon	T4N0M0
41	Female	66	ascending colon	T3N0M0
42	Male	62	rectum	T3N1M0
43	Male	46	rectum	T4N1M0
44	Female	69	hepatic flexure	T4N1M0

45	Male	64	hepatic flexure	T4N1M1
46	Male	79	ascending colon	T3N1M0
47	Male	67	rectum	T4N0M0
48	Male	63	splenic flexure	T3N0M0
49	Male	65	rectum	T2N0M0
50	Female	53	hepatic flexure	T4N0M0
51	Female	62	hepatic flexure	T4N1M0
52	Male	70	rectum	T4N1M0
53	Male	62	rectum	T2N0M0
54	Female	32	transverse colon	T3N0M0
55	Male	66	ascending colon	T3N2M0
56	Male	81	ileocecal junction	T4N0M0
57	Male	52	rectum	T4N3M0
58	Female	65	rectum	T3N2M1
59	Female	57	rectum	T4N1M0
60	Female	58	sigmoid colon	T3N2M0
61	Male	81	sigmoid colon	T4N1M0
62	Female	59	rectum	T4N2M1
63	Male	45	rectum	T4N2M0
64	Female	39	rectum	T1N0M0
65	Male	64	sigmoid colon	T4N1M0
66	Male	56	sigmoid colon	T2N1M1
67	Male	60	descending colon	T2N2M0
68	Male	69	rectum	T3N1M1
69	Female	64	sigmoid colon	T2N0M0
70	Male	67	ascending colon	T3N2M1
71	Male	61	descending colon	T4N0M1
72	Male	76	sigmoid colon	T4N0M0
73	Male	43	ascending colon	T3N1M0
74	Female	76	sigmoid colon	T4N0M1
75	Female	76	sigmoid colon	T3N2M1
76	Female	51	sigmoid colon	T4N1M0
77	Female	67	ascending colon	T2N0M0
78	Female	80	rectum	T2N1M0
79	Male	62	rectum	T3N0M0
80	Male	53	descending colon	T4N0M0
81	Male	79	ascending colon	T3N2M0
82	Male	69	sigmoid colon	T4N0M0
83	Male	72	rectum	T2N0M0
84	Male	58	ascending colon	T5N0M0
85	Male	57	sigmoid colon	T2N0M1
86	Female	67	transverse colon	T4N1M0
87	Male	69	sigmoid colon	T4N1M1
88	Male	41	rectum	T3N2M0
89	Male	87	ascending colon	T4N2M0
90	Female	26	sigmoid colon	T1N0M0

91	Male	62	rectum	T3N0M0
92	Male	56	rectum	T2N1M0
93	Male	56	rectum	T2N0M0
94	Male	59	rectum	T3N2M1
95	Female	54	ascending colon	T2N0M0
96	Male	55	rectum	T3N0M0
97	Male	71	ascending colon	T4N2M1
98	Male	60	sigmoid colon	T3N0M0
99	Male	67	descending colon	T4N0M0
100	Female	65	rectum	T3N2M1
101	Male	81	ascending colon	T3N0M0
102	Female	52	sigmoid colon	T2N0M0
103	Male	72	rectum	T3N2M1
104	Female	72	sigmoid colon	T4N0M0
105	Male	71	rectum	T3N0M0
106	Female	72	rectum	T4N1M0
107	Female	70	rectum	T4N0M0
108	Male	69	rectum	T3N1M0
109	Female	66	ascending colon	T4N0M0
110	Female	66	rectum	T4N0M0
111	Female	74	descending colon	T3N2M1
112	Female	64	ascending colon	T3N0M0
113	Female	76	sigmoid colon	T3N2M0
114	Male	68	rectum	T3N0M1
115	Female	51	rectum	T4N0M0
116	Female	49	ascending colon	T4N2M0
117	Male	79	ascending colon	T3N0M0
118	Male	63	rectum	T2N0M0
119	Male	58	sigmoid colon	T3N0M0
120	Female	56	ascending colon	T3N1M0
121	Female	63	sigmoid colon	T4N1M0
122	Male	63	transverse colon	T4N0M0
123	Male	63	descending colon	T3N0M0
124	Female	79	ascending colon	T4N0M0
125	Female	75	ascending colon	T3N0M0
126	Female	69	descending colon	T4N3M0
127	Male	44	rectum	T2N0M0
128	Male	69	sigmoid colon	T2N0M0
129	Male	58	rectum	T4N2M0
130	Male	62	rectum	T2N0M0
131	Male	66	ascending colon	T3N2M0
132	Female	58	rectum	T4N1M0
133	Female	63	sigmoid colon	T2N0M0
134	Male	70	ascending colon	T4N3M0
135	Female	75	sigmoid colon	T3N2M0
136	Female	61	sigmoid colon	T3N2M0

137	Male	82	sigmoid colon	T4N0M0
138	Female	48	rectum	T4N3M0
139	Female	57	ascending colon	T3N0M0
140	Male	63	rectum	T4N1M0
141	Male	67	descending colon	T2N0M0
142	Male	75	rectum	T3N0M0
143	Male	50	transverse colon	T3N0M0
144	Male	62	ascending colon	T3N1M1
145	Male	59	rectum	T4N0M0
146	Female	83	descending colon	T3N0M0
147	Female	63	ascending colon	T4N1M1
148	Male	72	ascending colon	T3N1M0
149	Male	69	rectum	T2N0M0
150	Male	64	sigmoid colon	T3N2M0
151	Male	54	sigmoid colon	T3N0M0
152	Male	50	sigmoid colon	T4N2M0
153	Male	77	rectum	T3N0M0
154	Male	62	ascending colon	T3N0M0
155	Male	46	ascending colon	T3N0M0
156	Male	47	rectum	T3N1M0
157	Male	60	rectum	T3N1M0
158	Female	53	rectum	T4N0M0
159	Male	66	ascending colon	T3N0M1
160	Male	70	sigmoid colon	T4N2M1
161	Male	59	sigmoid colon	T1N1M0
162	Male	73	ascending colon	T4N0M0
163	Male	55	ascending colon	T3N0M0
164	Male	61	rectum	T4N2M0
165	Female	59	sigmoid colon	T2N0M0
166	Male	57	rectum	T3N2M0
167	Male	63	ascending colon	T4N0M0
168	Female	49	sigmoid colon	T2N2M0
169	Female	52	rectum	T4N1M0
170	Female	58	rectum	T2N0M0
171	Male	55	rectum	T4N0M0
172	Male	72	sigmoid colon	T4N1M0
173	Male	74	descending colon	T3N0M0
174	Female	84	ascending colon	T4N2M0
175	Male	30	descending colon	T4N0M0
176	Male	62	rectum	T3N0M0
177	Female	69	rectum	T2N0M0
178	Male	68	ascending colon	T2N0M1
179	Male	86	descending colon	T1N0M0
180	Male	55	rectum	T2N0M0
181	Female	57	rectum	T4N1M0
182	Female	32	rectum	T4N1M0

183	Male	67	sigmoid colon	T4N2M0
184	Male	76	sigmoid colon	T2N0M0
185	Female	85	rectum	T3N0M0
186	Female	79	ascending colon	T4N0M0
187	Male	58	sigmoid colon	T4N1M0
188	Male	58	rectum	T2N0M0
189	Female	55	ascending colon	T3N0M0
190	Male	69	rectum	T3N0M0
191	Male	66	descending colon	T3N0M0
192	Male	43	rectum	T2N0M0
193	Female	63	descending colon	T2N0M0
194	Male	66	ascending colon	T3N1M0
195	Female	53	rectum	T3N0M0
196	Female	60	rectum	T4N0M0
197	Male	73	rectum	T2N0M0
198	Female	69	rectum	T4N0M0
199	Female	77	rectum	T4N2M0
200	Female	69	rectum	T4N0M0
201	Female	70	rectum	T2N1M0
202	Male	78	sigmoid colon	T4N1M0
203	Male	51	rectum	T2N1M0
204	Female	45	rectum	T4N1M0
205	Male	49	rectum	T4N0M0
206	Male	59	rectum	T2N0M0
207	Male	73	ascending colon	T3N0M0
208	Female	78	ascending colon	T4N1M0
209	Male	56	rectum	T4N2M1
210	Female	50	rectum	T1N0M0
211	Male	62	rectum	T4N2M1
212	Female	60	sigmoid colon	T3N0M0
213	Male	57	ascending colon	T4N0M0
214	Male	38	descending colon	T4N2M0
215	Male	62	sigmoid colon	T4N2M0
216	Male	57	rectum	T4N2M0
217	Male	55	rectum	T4N2M0
218	Male	67	rectum	T4N1M1
219	Male	78	sigmoid colon	T1N0M0
220	Female	65	sigmoid colon	T4N2M0
221	Male	64	sigmoid colon	T4N0M0
222	Male	62	rectum	T4N0M0
223	Male	64	rectum	T2N2M0
224	Male	79	rectum	T4N1M0
225	Male	67	sigmoid colon	T4N2M0
226	Male	65	ascending colon	T4N0M0
227	Male	51	rectum	T4N1M1
228	Female	69	rectum	T4N0M1

229	Female	79	ascending colon	T4N0M0
230	Female	64	ascending colon	T4N1M0
231	Male	62	sigmoid colon	T4N2M0
232	Male	70	descending colon	T4N1M0
233	Female	48	descending colon	T4N0M0
234	Male	78	sigmoid colon	T4N0M0
235	Female	51	rectum	T2N2M0
236	Female	83	rectum	T2N1M0
237	Female	74	ascending colon	T2N0M0
238	Male	81	ascending colon	T4N0M0
239	Male	81	rectum	T4N1M0
