# Supplementary material for: A 16q22.1 variant confers susceptibility to colorectal cancer as a distal regulator of ZFP90
Source: Oncogene. 2019 Oct 22;39(6):1347–60. doi: 10.1038/s41388-019-1055-4 (PMC7002302; doi:10.1038/s41388-019-1055-4)
Supplement: Supplementary file 8 — Table S2 [file 41388_2019_1055_MOESM8_ESM.pdf]

Table S2: Clinical information in Cohort 2 (FFPE)

Number	Status	OS(Months)	Gender	Age	Grade	Location	T	N	M	AJCC
1	Alive	69	Female	52	II	Proximal colon	T2	N0	M0	I
2	Alive	76	Female	51	II	Rectum	T1	N0	M0	I
3	Dead	40	Female	77	II	Distal colon	T2	N0	M0	I
4	Alive	69	Male	66	II	Distal colon	T2	N0	M0	I
5	Alive	74	Female	44	II	Proximal colon	T2	N0	M0	I
6	Alive	76	Female	59	II	Distal colon	T2	N0	M0	I
7	Alive	73	Male	79	II	Proximal colon	T3	N0	M0	II
8	Alive	75	Male	56	II	Proximal colon	T3	N0	M0	II
9	Alive	75	Male	85	II	Proximal colon	T3	N0	M0	II
10	Alive	77	Male	62	II	Proximal colon	T3	N0	M0	II
11	Alive	76	Male	73	II	Proximal colon	T3	N0	M0	II
12	Alive	70	Male	62	I	Proximal colon	T3	N0	M0	II
13	Alive	71	Male	43	I	Proximal colon	T3	N0	M0	II
14	Alive	71	Male	73	I	Proximal colon	T4a	N0	M0	II
15	Alive	72	Male	83	I	Proximal colon	T3	N0	M0	II
16	Dead	44	Female	50	II	Proximal colon	T4a	N0	M0	II
17	Alive	73	Female	65	II	Distal colon	T3	N0	M0	II
18	Alive	75	Male	73	II	Distal colon	T3	N0	M0	II
19	Alive	75	Female	59	II	Distal colon	T3	N0	M0	II
20	Alive	77	Male	78	II	Proximal colon	T3	N0	M0	II
21	Dead	13	Male	76	II	Distal colon	T3	N0	M0	II
22	Dead	38	Male	68	II	Proximal colon	T4a	N0	M0	II
23	Dead	35	Male	54	II	Proximal colon	T3	N0	M0	II
24	Alive	71	Female	46	II	Distal colon	T3	N0	M0	II
25	Alive	70	Male	73	II	Distal colon	T4a	N0	M0	II
26	Alive	75	Female	54	II	Distal colon	T3	N0	M0	II
27	Alive	73	Female	60	II	Distal colon	T3	N0	M0	II
28	Alive	77	Male	39	II	Proximal colon	T4a	N0	M0	II
29	Alive	77	Female	68	II	Proximal colon	T3	N0	M0	II
30	Dead	33	Female	79	II	Distal colon	T3	N0	M0	II
31	Dead	58	Female	47	II	Distal colon	T3	N0	M0	II
32	Alive	70	Male	62	II	Proximal colon	T3	N0	M0	II
33	Alive	70	Male	58	II	Proximal colon	T3	N0	M0	II
34	Alive	71	Male	83	II	Distal colon	T4a	N0	M0	II
35	Alive	72	Female	69	II	Proximal colon	T4a	N0	M0	II
36	Alive	78	Male	72	II	Proximal colon	T3	N0	M0	II
37	Alive	78	Female	63	II	Distal colon	T3	N0	M0	II
38	Dead	23	Female	62	II	Proximal colon	T4a	N1b	M0	III
39	Alive	69	Female	81	II	Proximal colon	T4a	N1a	M0	III
40	Alive	69	Female	55	II	Distal colon	T4a	N1b	M0	III
41	Dead	13	Female	76	II	Distal colon	T4a	N1b	M0	III
42	Dead	19	Male	65	II	Distal colon	T3	N2a	M0	III
43	Alive	75	Male	81	II	Distal colon	T3	N1b	M0	III
44	Dead	16	Male	65	III	Proximal colon	T4b	N1b	M0	III
45	Dead	7	Male	63	III	Distal colon	T4a	N2b	M0	III
46	Alive	71	Female	69	II	Proximal colon	T2	N0	M0	I

47	Alive	73	Male	61	II	Distal colon	T2	N0	M0	I
48	Alive	77	Female	83	I	Distal colon	T2	N0	M0	I
49	Alive	75	Female	70	II	Distal colon	T2	N0	M0	I
50	Alive	75	Female	65	II	Proximal colon	T3	N0	M0	II
51	Alive	74	Female	76	II	Proximal colon	T3	N0	M0	II
52	Alive	75	Male	79	II	Distal colon	T3	N0	M0	II
53	Dead	8	Female	52	III	Distal colon	T4a	N0	M0	II
54	Alive	72	Female	55	III	Proximal colon	T3	N0	M0	II
55	Dead	12	Female	48	III	Proximal colon	T3	N0	M0	II
56	Dead	67	Female	78	II	Distal colon	T3	N0	M0	II
57	Alive	70	Female	81	II	Proximal colon	T4a	N0	M0	II
58	Alive	73	Male	75	II	Proximal colon	T3	N0	M0	II
59	Alive	75	Male	80	II	Proximal colon	T3	N0	M0	II
60	Alive	75	Female	60	II	Distal colon	T3	N0	M0	II
61	Dead	1	Female	48	II	Proximal colon	T3	N0	M0	II
62	Dead	21	Male	55	II	Distal colon	T4a	N0	M0	II
63	Dead	40	Male	90	II	Distal colon	T4a	N0	M0	II
64	Alive	71	Male	57	II	Proximal colon	T3	N0	M0	II
65	Alive	70	Female	50	II	Proximal colon	T3	N0	M0	II
66	Alive	76	Female	61	II	Proximal colon	T3	N0	M0	II
67	Dead	13	Male	73	II	Distal colon	T3	N0	M0	II
68	Dead	1	Female	57	II	Proximal colon	T2	N1b	M0	III
69	Dead	13	Female	87	II	Proximal colon	T4b	N1b	M0	III
70	Dead	39	Male	63	II	Distal colon	T4a	N1b	M0	III
71	Alive	69	Female	77	II	Distal colon	T4a	N1a	M0	III
72	Alive	71	Female	82	III	Proximal colon	T3	N1a	M0	III
73	Dead	23	Female	66	III	Proximal colon	T4b	N1b	M0	III
74	Alive	72	Female	80	III	Proximal colon	T4a	N1a	M0	III
75	Alive	73	Male	55	III	Proximal colon	T4a	N1b	M0	III
76	Alive	76	Male	31	III	Proximal colon	T3	N1b	M0	III
77	Dead	15	Male	56	II	Distal colon	T3	N1b	M0	III
78	Alive	73	Male	81	II	Distal colon	T3	N1a	M0	III
79	Alive	77	Female	78	II	Proximal colon	T3	N1a	M0	III
80	Dead	42	Male	78	II	Proximal colon	T3	N1a	M0	III
81	Dead	42	Male	60	II	Distal colon	T3	N2b	M0	III
82	Dead	56	Male	55	II	Distal colon	T4a	N2a	M0	III
83	Dead	67	Male	54	II	Distal colon	T4a	N1b	M0	III
84	Dead	7	Female	73	II	Proximal colon	T3	N1a	M0	III
85	Dead	19	Male	67	II	Distal colon	T3	N1a	M0	III
86	Dead	0.4	Male	82	III	Distal colon	T3	N2b	M0	III
87	Dead	19	Female	27	III	Distal colon	T4a	N2a	M0	III
88	Dead	22	Male	78	II	Distal colon	T3	N0	M1b	IV
89	Dead	17	Male	73	III	Proximal colon	T4a	N0	M1b	IV
90	Dead	16	Male	61	III	Rectum	T4a	N2a	M1a	IV
