# Supplementary material for: A 16q22.1 variant confers susceptibility to colorectal cancer as a distal regulator of ZFP90
Source: Oncogene. 2019 Oct 22;39(6):1347–60. doi: 10.1038/s41388-019-1055-4 (PMC7002302; doi:10.1038/s41388-019-1055-4)
Supplement: Supplementary file 9 — Table S3 [file 41388_2019_1055_MOESM9_ESM.pdf]

Table S3: RNA-sequence analysis in WT and ZFP90-KO HCT116 cells (q<0.05, log2(fold_change)>1 or <-1)

gene_id	gene	locus	value_1	value_2	log2(fold_c	test_stat	p_value	q_value	significant
XLOC_000	TRNP1	1:2732019	13.7999	60.1707	2.1244	11.1018	5.00E-05	0.001069	yes
XLOC_000	PRKAA2	1:5711099	3.97145	1.25084	-1.66677	-8.65043	5.00E-05	0.001069	yes
XLOC_001	RP11-435E	1:1433479	0.601	0.05827	-3.36652	-0.35262	5.00E-05	0.001069	yes
XLOC_001	PBX1	1:1645248	19.0906	6.50141	-1.55404	-5.25907	5.00E-05	0.001069	yes
XLOC_002	TROVE2	1:1929813	5.7908	21.1597	1.86949	4.54447	5.00E-05	0.001069	yes
XLOC_002	CDC73	1:1930911	7.12512	14.9814	1.07219	6.28662	5.00E-05	0.001069	yes
XLOC_002	C1orf106	1:2008601	1.25513	10.9582	3.1261	4.90439	5.00E-05	0.001069	yes
XLOC_002	ZBTB18	1:2442145	0.69518	1.41458	1.02492	3.45264	5.00E-05	0.001069	yes
XLOC_002	RP11-54O7	1:852244-	5.63531	2.786	-1.0163	-4.28845	5.00E-05	0.001069	yes
XLOC_002	TNFRSF9	1:7979906	1.95155	0.429267	-2.18468	-2.28619	5.00E-05	0.001069	yes
XLOC_002	TCEA3	1:2370755	0.37602	1.67872	2.15847	2.0995	5.00E-05	0.001069	yes
XLOC_002	ID3	1:2388440	166.893	59.8612	-1.47923	-9.18682	5.00E-05	0.001069	yes
XLOC_003	SLC2A1	1:4339105	130.426	52.178	-1.32171	-6.90095	5.00E-05	0.001069	yes
XLOC_003	PPAP2B	1:5696041	3.79991	1.82139	-1.06093	-2.86854	5.00E-05	0.001069	yes
XLOC_003	TACSTD2	1:5904109	11.8318	29.2947	1.30797	7.22618	5.00E-05	0.001069	yes
XLOC_003	ARHGAP29	1:9461454	27.2782	81.0101	1.57036	9.45564	5.00E-05	0.001069	yes
XLOC_003	F3	1:9499478	25.1394	89.0413	1.82452	10.3037	5.00E-05	0.001069	yes
XLOC_003	EPS8L3	1:1102548	2.8795	0.178059	-4.01539	-1.1843	5.00E-05	0.001069	yes
XLOC_004	SPRR2D	1:1530122	18.241	7.00878	-1.37995	-5.02357	5.00E-05	0.001069	yes
XLOC_004	IQGAP3	1:1564951	9.71835	4.54246	-1.09724	-2.73995	5.00E-05	0.001069	yes
XLOC_004	NES	1:1566385	0.91796	2.05616	1.16344	5.2971	5.00E-05	0.001069	yes
XLOC_004	TNFSF4	1:1731528	2.18109	0.715515	-1.60799	-4.0596	5.00E-05	0.001069	yes
XLOC_004	RGS16	1:1825677	16.5436	8.21467	-1.01	-5.61748	5.00E-05	0.001069	yes
XLOC_004	UCHL5	1:1929813	14.6905	37.8579	1.36571	5.00723	5.00E-05	0.001069	yes
XLOC_004	GLRX2	1:1930655	12.4812	41.6468	1.73845	7.79458	5.00E-05	0.001069	yes
XLOC_004	LMOD1	1:2018629	0.44738	1.19233	1.41422	4.51151	5.00E-05	0.001069	yes
XLOC_004	LEMD1	1:2053423	4.19271	9.92726	1.24351	3.51864	5.00E-05	0.001069	yes
XLOC_004	C1orf116	1:2071918	1.53472	3.4814	1.18169	4.3818	5.00E-05	0.001069	yes
XLOC_005	FXYD4	10:438670	13.8121	4.14981	-1.73482	-3.08752	5.00E-05	0.001069	yes
XLOC_005	ALOX5	10:458696	5.08041	1.47005	-1.78908	-2.29952	5.00E-05	0.001069	yes
XLOC_005	COL13A1	10:715616	1.3487	5.16409	1.93695	4.44269	5.00E-05	0.001069	yes
XLOC_005	PLAU	10:756689	53.2052	166.291	1.64407	9.66094	5.00E-05	0.001069	yes
XLOC_005	ABCC2	10:101542	119.482	35.632	-1.74555	-6.30514	5.00E-05	0.001069	yes
XLOC_006	TUBAL3	10:543506	1.53808	0.477904	-1.68634	-3.38683	5.00E-05	0.001069	yes
XLOC_006	NEBL	10:210689	23.4847	11.3037	-1.05493	-4.39758	5.00E-05	0.001069	yes
XLOC_006	CASC10	10:217815	0.68449	1.64521	1.26516	3.9217	5.00E-05	0.001069	yes
XLOC_006	SVIL	10:296983	12.6824	5.6841	-1.15782	-2.53304	5.00E-05	0.001069	yes
XLOC_006	ZEB1-AS1	10:314768	2.23648	4.54259	1.02228	1.84487	5.00E-05	0.001069	yes
XLOC_006	NRP1	10:334664	1.37258	3.88236	1.50004	3.01772	5.00E-05	0.001069	yes
XLOC_006	NAMPTL	10:368106	0.20592	1.46161	2.82738	3.04222	5.00E-05	0.001069	yes
XLOC_006	PRF1	10:723571	5.57771	1.69319	-1.71993	-6.63652	5.00E-05	0.001069	yes
XLOC_006	HTR7	10:925005	33.0631	14.0367	-1.23601	-7.54661	5.00E-05	0.001069	yes
XLOC_007	ADM	11:103262	7.8446	16.4856	1.07144	3.96816	5.00E-05	0.001069	yes
XLOC_007	SERPING1	11:573648	0.21595	1.0246	2.24627	1.07991	5.00E-05	0.001069	yes
XLOC_008	CDC42EP2	11:650822	5.36365	11.5023	1.10063	4.97213	5.00E-05	0.001069	yes
XLOC_008	C11orf86	11:667427	2.4627	0.560431	-2.13564	-3.2409	5.00E-05	0.001069	yes
XLOC_008	SLC37A2	11:124932	1.48	3.19798	1.11157	2.53382	5.00E-05	0.001069	yes
XLOC_008	H19	11:201640	4.36112	1.9043	-1.19544	-1.64534	5.00E-05	0.001069	yes

XLOC_008	PRKCDBP	11:634017	2.34836	7.64981	1.70377	4.05005	5.00E-05	0.001069	yes
XLOC_009	MRVI1	11:105628	0.50354	1.5117	1.58601	1.46973	5.00E-05	0.001069	yes
XLOC_009	CDCA5	11:648337	16.1996	7.47496	-1.11582	-5.13286	5.00E-05	0.001069	yes
XLOC_009	PDE2A	11:722871	6.68886	15.0999	1.17471	2.6989	5.00E-05	0.001069	yes
XLOC_009	SYTL2	11:854052	7.87036	3.93159	-1.00132	-2.4025	5.00E-05	0.001069	yes
XLOC_009	SESN3	11:948837	2.07982	0.816568	-1.34881	-2.87667	5.00E-05	0.001069	yes
XLOC_010	TRIM29	11:119981	1.36236	0.404638	-1.75141	-0.7841	5.00E-05	0.001069	yes
XLOC_010	ETS1	11:128328	2.09292	4.37043	1.06226	4.06759	5.00E-05	0.001069	yes
XLOC_010	METTL7A	12:513172	1.65191	0.486862	-1.76255	-2.5437	5.00E-05	0.001069	yes
XLOC_011	TPH2,TRHI	12:723326	0.05436	0.612979	3.49516	0.44326	5.00E-05	0.001069	yes
XLOC_011	ELK3	12:965881	13.9704	32.2127	1.20526	5.99431	5.00E-05	0.001069	yes
XLOC_011	P2RX7	12:121570	2.61498	0.49114	-2.41259	-3.17081	5.00E-05	0.001069	yes
XLOC_011	ARHGDIB	12:150949	0.96731	0.141879	-2.76931	-0.7748	5.00E-05	0.001069	yes
XLOC_012	LIMA1	12:505695	22.388	45.5359	1.02428	4.68748	5.00E-05	0.001069	yes
XLOC_012	RP11-230C	12:656724	1.11264	4.69933	2.07847	2.72593	5.00E-05	0.001069	yes
XLOC_012	RP11-13A1	12:894044	0.79466	2.19889	1.46837	5.06026	5.00E-05	0.001069	yes
XLOC_012	DCN	12:915390	0.18838	0.987718	2.39046	0.57981	5.00E-05	0.001069	yes
XLOC_012	FGD6	12:954705	5.18111	10.4172	1.00763	4.20332	5.00E-05	0.001069	yes
XLOC_012	ALDH1L2	12:105380	0.17625	1.006	2.51291	1.56913	5.00E-05	0.001069	yes
XLOC_012	TBX3	12:115108	14.676	3.10939	-2.23876	-9.44603	5.00E-05	0.001069	yes
XLOC_012	HPD	12:122277	3.61243	0.363057	-3.3147	-2.94094	5.00E-05	0.001069	yes
XLOC_013	DGKH	13:426141	1.09623	2.41936	1.14207	2.43482	5.00E-05	0.001069	yes
XLOC_013	FOXO1	13:411298	9.83595	4.43214	-1.15006	-5.05953	5.00E-05	0.001069	yes
XLOC_014	PELI2	14:565845	1.54413	0.553495	-1.48015	-2.24077	5.00E-05	0.001069	yes
XLOC_014	DACT1	14:591006	0.21894	1.49075	2.76745	2.44247	5.00E-05	0.001069	yes
XLOC_014	KCNK13	14:905281	0.55163	1.19406	1.11411	2.5234	5.00E-05	0.001069	yes
XLOC_014	PPP4R4	14:946124	1.609	3.47287	1.10996	2.30247	5.00E-05	0.001069	yes
XLOC_014	RP11-986E	14:950274	2.95937	6.52152	1.13992	3.11984	5.00E-05	0.001069	yes
XLOC_014	TCL1B	14:961168	0.10631	0.86809	3.02961	0.99219	5.00E-05	0.001069	yes
XLOC_015	RN7SL2	14:503292	513.685	227.209	-1.17686	-6.05108	5.00E-05	0.001069	yes
XLOC_015	AL162759.	14:542356	23.1106	2.08856	-3.46797	-8.01139	5.00E-05	0.001069	yes
XLOC_015	BMP4	14:544164	230.724	15.557	-3.89053	-17.9199	5.00E-05	0.001069	yes
XLOC_015	STON2	14:817269	2.4638	0.792001	-1.63731	-2.08619	5.00E-05	0.001069	yes
XLOC_015	SERPINA1	14:948430	1.18127	3.57581	1.59794	2.29805	5.00E-05	0.001069	yes
XLOC_015	LINC00341	14:958736	0.80184	2.09175	1.38332	4.47187	5.00E-05	0.001069	yes
XLOC_015	SYNE3	14:958838	1.03907	2.70108	1.37824	4.9417	5.00E-05	0.001069	yes
XLOC_016	CASC5	15:408862	4.74639	2.11557	-1.16578	-2.23026	5.00E-05	0.001069	yes
XLOC_017	ALDH1A3	15:101389	28.5987	78.3112	1.45327	4.3545	5.00E-05	0.001069	yes
XLOC_017	STRA6	15:744718	0.33047	1.21845	1.88244	1.04046	5.00E-05	0.001069	yes
XLOC_018	RP11-66B2	15:101389	14.3964	38.8005	1.43037	4.36728	5.00E-05	0.001069	yes
XLOC_018	C16orf52	16:220189	12.6803	25.8205	1.02593	3.60142	5.00E-05	0.001069	yes
XLOC_018	MT2A	16:566421	312.814	704.523	1.17134	7.4436	5.00E-05	0.001069	yes
XLOC_018	MT1E	16:566593	252.881	569.433	1.17107	6.27797	5.00E-05	0.001069	yes
XLOC_018	MT1X	16:567163	37.2492	102.289	1.45736	6.2135	5.00E-05	0.001069	yes
XLOC_019	CALB2	16:713926	9.2263	20.8818	1.17842	5.65311	5.00E-05	0.001069	yes
XLOC_019	ABCC6	16:162427	0.26345	0.89122	1.75823	1.54507	5.00E-05	0.001069	yes
XLOC_019	PRSS8	16:311270	11.2397	37.2437	1.72839	4.41447	5.00E-05	0.001069	yes
XLOC_020	ENKD1	16:676968	3.83106	9.98801	1.38245	3.39218	5.00E-05	0.001069	yes
XLOC_020	GRB7	17:378941	19.0454	40.5223	1.08928	4.83263	5.00E-05	0.001069	yes
XLOC_021	MRC2	17:607047	4.12829	9.8877	1.26009	4.08808	5.00E-05	0.001069	yes

XLOC_022	KRT20	17:390321	3.09059	0.567551	-2.44506	-3.51166	5.00E-05	0.001069	yes
XLOC_022	KRT23	17:390776	23.2642	10.7785	-1.10995	-5.6156	5.00E-05	0.001069	yes
XLOC_022	KRT13	17:396572	14.4905	1.3309	-3.44463	-5.24741	5.00E-05	0.001069	yes
XLOC_022	KRT16	17:397660	8.18198	2.8647	-1.51407	-5.64393	5.00E-05	0.001069	yes
XLOC_022	SKAP1	17:462108	2.08922	4.97272	1.25107	3.4838	5.00E-05	0.001069	yes
XLOC_022	HOXB3,HC	17:466182	14.1153	33.4399	1.24431	2.94706	5.00E-05	0.001069	yes
XLOC_022	SMURF2	17:624957	33.1759	89.1885	1.42672	2.65364	5.00E-05	0.001069	yes
XLOC_022	AC005152	17:700179	0.49047	2.00129	2.02869	0.89653	5.00E-05	0.001069	yes
XLOC_022	SECTM1	17:802788	3.25311	6.67052	1.03598	2.08106	5.00E-05	0.001069	yes
XLOC_023	CYP4F35P	18:143374	2.51331	0.631076	-1.99371	-3.18776	5.00E-05	0.001069	yes
XLOC_023	GATA6	18:197494	1.18259	2.55322	1.11036	3.23467	5.00E-05	0.001069	yes
XLOC_023	LAMA3	18:212694	1.65225	3.64241	1.14046	2.4716	5.00E-05	0.001069	yes
XLOC_023	DSC3	18:285699	0.23378	3.52435	3.91416	7.31943	5.00E-05	0.001069	yes
XLOC_023	CCDC68	18:525687	3.87177	8.36707	1.11173	2.99325	5.00E-05	0.001069	yes
XLOC_024	CYP4F12	19:157835	9.46163	3.10127	-1.60923	-3.89279	5.00E-05	0.001069	yes
XLOC_024	PAPL	19:395745	2.58635	6.12137	1.24294	4.62334	5.00E-05	0.001069	yes
XLOC_025	ZNF701,ZN	19:530309	56.4958	5.28263	-3.41881	-12.8314	5.00E-05	0.001069	yes
XLOC_025	NLRP2	19:554348	35.7238	17.0579	-1.06644	-3.77948	5.00E-05	0.001069	yes
XLOC_025	AC003005	19:579990	2.44792	1.05994	-1.20757	-1.2397	5.00E-05	0.001069	yes
XLOC_025	ADAMTS10	19:864512	6.21698	12.5401	1.01226	2.80464	5.00E-05	0.001069	yes
XLOC_025	UNC13A	19:177121	8.81281	21.8046	1.30696	4.18491	5.00E-05	0.001069	yes
XLOC_026	NLRP12	19:542968	1.1263	0.310434	-1.85923	-3.38647	5.00E-05	0.001069	yes
XLOC_026	COX6B2,C1	19:558606	27.3127	1.80024	-3.92331	-7.43988	5.00E-05	0.001069	yes
XLOC_026	NLRP11	19:562967	2.03094	0.912571	-1.15414	-2.91078	5.00E-05	0.001069	yes
XLOC_026	RRM2	2:1026245	41.57	20.4946	-1.0203	-5.25774	5.00E-05	0.001069	yes
XLOC_026	FOSL2	2:2860727	23.9933	11.9262	-1.00851	-5.98367	5.00E-05	0.001069	yes
XLOC_027	SULT1C2	2:1089050	10.8961	2.05113	-2.40932	-6.34115	5.00E-05	0.001069	yes
XLOC_027	GLI2	2:1214931	0.22593	1.07488	2.25023	1.15515	5.00E-05	0.001069	yes
XLOC_027	GALNT5	2:1581141	0.46852	2.07152	2.14451	3.83	5.00E-05	0.001069	yes
XLOC_027	H3F3AP4	2:1754118	8.56575	19.793	1.20834	5.24738	5.00E-05	0.001069	yes
XLOC_028	BZW1	2:2015429	94.7765	191.904	1.01778	5.84476	5.00E-05	0.001069	yes
XLOC_028	CASP10	2:2020476	1.23829	2.49864	1.01279	1.80811	5.00E-05	0.001069	yes
XLOC_028	SGPP2	2:2232892	6.3847	2.12354	-1.58815	-7.10935	5.00E-05	0.001069	yes
XLOC_028	MLPH	2:2383940	41.7817	88.6789	1.08572	5.03261	5.00E-05	0.001069	yes
XLOC_028	RAMP1	2:2387675	5.6881	19.4079	1.77063	6.10919	5.00E-05	0.001069	yes
XLOC_028	PXDN	2:1635658	2.76654	6.4186	1.21417	2.20042	5.00E-05	0.001069	yes
XLOC_028	MBOAT2	2:8992819	3.17283	7.93499	1.32246	2.09484	5.00E-05	0.001069	yes
XLOC_029	STK39	2:1688105	42.8215	101.352	1.24297	6.09841	5.00E-05	0.001069	yes
XLOC_029	DLX2	2:1729641	3.7105	1.57688	-1.23454	-3.78337	5.00E-05	0.001069	yes
XLOC_030	ABCA12	2:2156749	2.57598	0.958752	-1.42589	-3.60126	5.00E-05	0.001069	yes
XLOC_030	EPHA4	2:2222827	2.75674	1.06023	-1.37858	-1.42679	5.00E-05	0.001069	yes
XLOC_030	AC068138	2:2270447	4.00475	1.63231	-1.2948	-3.89024	5.00E-05	0.001069	yes
XLOC_030	ARL4C	2:2354016	1.20331	2.8644	1.25123	5.01044	5.00E-05	0.001069	yes
XLOC_030	PER2	2:2391403	1.97896	4.7155	1.25267	3.135	5.00E-05	0.001069	yes
XLOC_030	RP11-290F	20:489092	2.47301	5.33157	1.1083	2.16276	5.00E-05	0.001069	yes
XLOC_031	CYP24A1	20:527699	81.1518	180.227	1.15112	4.53448	5.00E-05	0.001069	yes
XLOC_031	B3GALT5	21:409283	0.51974	2.91667	2.48846	6.87781	5.00E-05	0.001069	yes
XLOC_032	TIAM1	21:324907	8.52424	24.6161	1.52996	5.16861	5.00E-05	0.001069	yes
XLOC_032	SIK1	21:448343	19.1008	6.92721	-1.46329	-6.68511	5.00E-05	0.001069	yes
XLOC_032	LINC00896	22:201939	1.45549	2.97451	1.03115	3.29609	5.00E-05	0.001069	yes

XLOC_032	CYTH4	22:376780	0.64075	2.52866	1.98054	1.73443	5.00E-05	0.001069	yes
XLOC_032	TEF	22:417633	4.00414	8.48008	1.08259	5.34081	5.00E-05	0.001069	yes
XLOC_033	PRAME	22:228901	4.44959	15.2294	1.77512	6.91391	5.00E-05	0.001069	yes
XLOC_033	MB	22:360028	2.22001	5.05737	1.18782	2.17932	5.00E-05	0.001069	yes
XLOC_033	PDGFB	22:396193	4.78562	13.3911	1.4845	5.69601	5.00E-05	0.001069	yes
XLOC_033	MYRIP	3:3985040	0.61596	0.084182	-2.87126	-1.20737	5.00E-05	0.001069	yes
XLOC_035	OSBPL10	3:3169938	3.4037	7.48972	1.13781	1.65736	5.00E-05	0.001069	yes
XLOC_035	KLF15	3:1260614	1.40737	2.91928	1.05261	2.78501	5.00E-05	0.001069	yes
XLOC_035	MGLL	3:1274079	3.85619	11.223	1.54121	3.94358	5.00E-05	0.001069	yes
XLOC_035	GATA2	3:1281982	12.0802	4.71217	-1.35817	-3.93315	5.00E-05	0.001069	yes
XLOC_036	TNIK	3:1707791	3.96458	9.91015	1.32174	3.50577	5.00E-05	0.001069	yes
XLOC_036	CPEB2	4:1500429	1.54565	0.562022	-1.45952	-1.9761	5.00E-05	0.001069	yes
XLOC_036	SLIT2	4:2025488	2.73275	0.942537	-1.53573	-2.0106	5.00E-05	0.001069	yes
XLOC_037	IL8	4:7460622	8.77814	4.31776	-1.02363	-4.19576	5.00E-05	0.001069	yes
XLOC_037	EPGN	4:7517418	4.39551	0.699248	-2.65216	-1.92096	5.00E-05	0.001069	yes
XLOC_037	ANXA10	4:1688678	85.6223	10.3507	-3.04825	-12.9333	5.00E-05	0.001069	yes
XLOC_037	PALLD	4:1692778	66.8431	33.1702	-1.01089	-2.73532	5.00E-05	0.001069	yes
XLOC_037	FGFBP1	4:1593719	14.8019	5.10238	-1.53654	-6.92951	5.00E-05	0.001069	yes
XLOC_038	INPP4B	4:1429443	2.40087	8.00536	1.73741	4.1705	5.00E-05	0.001069	yes
XLOC_038	PDGFC	4:1576816	3.9581	8.74444	1.14356	3.37786	5.00E-05	0.001069	yes
XLOC_039	SLC1A3	5:3660645	1.21216	6.74417	2.47606	3.23241	5.00E-05	0.001069	yes
XLOC_039	RHOBTB3	5:9503695	14.6649	36.0534	1.29776	3.94108	5.00E-05	0.001069	yes
XLOC_039	LYRM7	5:1304947	2.69713	6.80257	1.33466	1.7512	5.00E-05	0.001069	yes
XLOC_040	STK32A	5:1466145	5.23668	0.75966	-2.78522	-3.79619	5.00E-05	0.001069	yes
XLOC_040	AFAP1L1	5:1485210	0.79867	2.1298	1.41505	1.89508	5.00E-05	0.001069	yes
XLOC_040	FABP6	5:1596143	8.17653	3.6921	-1.14705	-3.27519	5.00E-05	0.001069	yes
XLOC_040	RP11-43F1	5:987294-	0.92764	0.430123	-1.10882	-2.5808	5.00E-05	0.001069	yes
XLOC_040	PDE4D	5:5826486	0.1062	0.938884	3.14412	0.53097	5.00E-05	0.001069	yes
XLOC_041	DPYSL3	5:1467703	7.10885	3.16192	-1.16882	-4.02132	5.00E-05	0.001069	yes
XLOC_041	DRD1	5:1748670	0.77247	1.58028	1.03263	3.6869	5.00E-05	0.001069	yes
XLOC_041	SOX4	6:2159397	56.3127	17.0374	-1.72475	-10.8181	5.00E-05	0.001069	yes
XLOC_042	TPBG	6:8307292	56.2383	26.0346	-1.11113	-6.78341	5.00E-05	0.001069	yes
XLOC_042	NT5E	6:8615980	41.6623	88.2892	1.08349	6.25096	5.00E-05	0.001069	yes
XLOC_042	TMEM200	6:1306868	2.4726	6.50649	1.39585	6.45922	5.00E-05	0.001069	yes
XLOC_043	PPP1R14C	6:1504642	2.89665	7.02711	1.27855	5.5507	5.00E-05	0.001069	yes
XLOC_043	C6orf1	6:3421415	4.10621	9.97381	1.28034	3.81247	5.00E-05	0.001069	yes
XLOC_043	FKBP5	6:3554136	9.63485	19.3638	1.00703	6.0597	5.00E-05	0.001069	yes
XLOC_043	MDGA1	6:3760028	0.49088	2.51009	2.3543	1.83639	5.00E-05	0.001069	yes
XLOC_043	GPR110	6:4696543	2.5974	11.8038	2.18411	6.15845	5.00E-05	0.001069	yes
XLOC_043	COL12A1	6:7579404	2.27991	5.49123	1.26815	3.9414	5.00E-05	0.001069	yes
XLOC_043	IBTK	6:8287969	67.3399	32.3884	-1.05599	-6.73409	5.00E-05	0.001069	yes
XLOC_044	BVES	6:1055446	0.34986	0.871682	1.31703	2.18951	5.00E-05	0.001069	yes
XLOC_044	POPDC3	6:1055446	4.5691	12.3738	1.4373	4.57336	5.00E-05	0.001069	yes
XLOC_044	LAMA4	6:1124299	0.1049	0.714035	2.76698	0.45832	5.00E-05	0.001069	yes
XLOC_044	MAN1A1	6:1194983	52.4858	17.9191	-1.55043	-9.94465	5.00E-05	0.001069	yes
XLOC_044	CTGF	6:1322693	0.31882	1.03832	1.70342	3.21918	5.00E-05	0.001069	yes
XLOC_044	RP3-323P1	6:1335617	0.50365	3.83678	2.92939	2.51715	5.00E-05	0.001069	yes
XLOC_044	ITGB8	7:2036792	0.19685	1.03123	2.38918	3.73548	5.00E-05	0.001069	yes
XLOC_044	HOTAIRM1	7:2713261	5.61107	18.0795	1.68801	4.83303	5.00E-05	0.001069	yes
XLOC_044	HOXA-AS2	7:2714580	0.47182	2.35469	2.31921	1.49905	5.00E-05	0.001069	yes

XLOC_045	SERPINE1	7:1007703	7.18155	24.1003	1.74668	10.2646	5.00E-05	0.001069	yes
XLOC_045	PRKAR2B	7:1066850	1.58333	3.31707	1.06695	3.25564	5.00E-05	0.001069	yes
XLOC_045	FEZF1-AS1	7:1219414	1.81896	4.27431	1.23258	2.47331	5.00E-05	0.001069	yes
XLOC_045	CCDC136	7:1284308	2.00075	5.62584	1.49153	2.46859	5.00E-05	0.001069	yes
XLOC_045	FLNC	7:1284704	10.3158	25.5542	1.3087	7.27584	5.00E-05	0.001069	yes
XLOC_045	SSPO	7:1494731	1.75575	5.53719	1.65707	2.89193	5.00E-05	0.001069	yes
XLOC_046	DFNA5	7:2473797	10.5499	5.26467	-1.00281	-3.04219	5.00E-05	0.001069	yes
XLOC_046	SKAP2	7:2670668	4.97301	14.2406	1.51782	3.11404	5.00E-05	0.001069	yes
XLOC_046	HOXA1	7:2713261	0.69525	2.06836	1.57288	3.23606	5.00E-05	0.001069	yes
XLOC_046	HOXA3,HC	7:2714580	3.3048	11.1855	1.759	3.24133	5.00E-05	0.001069	yes
XLOC_046	SUN3	7:4802674	6.29099	18.3364	1.54335	5.19409	5.00E-05	0.001069	yes
XLOC_046	SEMA3C	7:8037185	18.3889	8.57092	-1.10132	-4.39284	5.00E-05	0.001069	yes
XLOC_046	SEMA3A	7:8358509	21.0899	2.57735	-3.03259	-6.09737	5.00E-05	0.001069	yes
XLOC_046	CDK6	7:9223423	2.40756	7.07149	1.55444	4.54994	5.00E-05	0.001069	yes
XLOC_046	ACHE	7:1004876	0.52502	1.69926	1.69446	1.12433	5.00E-05	0.001069	yes
XLOC_046	VGF	7:1008057	13.1487	46.5924	1.82517	10.365	5.00E-05	0.001069	yes
XLOC_047	ZNF467	7:1494612	4.18008	10.7606	1.36416	5.11015	5.00E-05	0.001069	yes
XLOC_047	SLC7A2	8:1735459	2.11551	4.27211	1.01395	5.35922	5.00E-05	0.001069	yes
XLOC_047	NRG1	8:3149690	0.11898	0.971997	3.03027	0.83859	5.00E-05	0.001069	yes
XLOC_047	LETM2	8:3824372	5.05804	13.5169	1.41812	3.86305	5.00E-05	0.001069	yes
XLOC_048	SQLE	8:1260107	2.66754	18.8176	2.8185	10.3959	5.00E-05	0.001069	yes
XLOC_048	RP11-909N	8:1444998	3.29016	12.1301	1.88237	3.569	5.00E-05	0.001069	yes
XLOC_048	RP11-350N	8:3824372	3.06228	7.1444	1.22221	2.42119	5.00E-05	0.001069	yes
XLOC_048	SDR16C5	8:5721256	2.09696	0.912453	-1.20048	-3.1752	5.00E-05	0.001069	yes
XLOC_049	TMEM55A	8:9200602	3.64185	7.64445	1.06974	2.89033	5.00E-05	0.001069	yes
XLOC_049	LY6D	8:1438662	7.60782	1.23807	-2.6194	-4.76194	5.00E-05	0.001069	yes
XLOC_049	RP11-111(	9:4029302	0.41598	0.833896	1.00335	2.37253	5.00E-05	0.001069	yes
XLOC_050	C9orf3	9:9748898	6.66534	2.44516	-1.44675	-2.37849	5.00E-05	0.001069	yes
XLOC_050	OLFML2A	9:1275394	0.62076	1.55369	1.3236	3.63653	5.00E-05	0.001069	yes
XLOC_050	LCN2	9:1309113	46.88	113.958	1.28146	6.33136	5.00E-05	0.001069	yes
XLOC_050	LCNL1,PTG	9:1398719	0.58992	3.26986	2.47064	1.52961	5.00E-05	0.001069	yes
XLOC_050	GLIS3	9:3824126	0.51003	1.31001	1.36092	1.13972	5.00E-05	0.001069	yes
XLOC_050	NFIB	9:1408184	0.43833	1.96201	2.16225	2.6056	5.00E-05	0.001069	yes
XLOC_051	RP11-381(	9:6701739	0.49064	1.57828	1.68563	2.35022	5.00E-05	0.001069	yes
XLOC_051	PGM5P2,R	9:6906565	0.52226	1.6566	1.6654	2.67473	5.00E-05	0.001069	yes
XLOC_051	FRMD3	9:8585790	1.01442	0.330448	-1.61816	-1.70684	5.00E-05	0.001069	yes
XLOC_051	STOM	9:1241013	6.27262	12.6849	1.01597	3.84916	5.00E-05	0.001069	yes
XLOC_057	PLCXD1	X:192988-	20.8713	9.00239	-1.21314	-3.068	5.00E-05	0.001069	yes
XLOC_057	CSF2RA	X:1387692	6.88431	2.85567	-1.26948	-2.78599	5.00E-05	0.001069	yes
XLOC_057	CHST7	X:4643321	2.26263	5.07622	1.16576	4.08271	5.00E-05	0.001069	yes
XLOC_058	RNF128	X:1059370	1.36512	3.16271	1.21213	4.20246	5.00E-05	0.001069	yes
XLOC_058	FHL1	X:1352295	8.47882	21.0277	1.31036	6.29444	5.00E-05	0.001069	yes
XLOC_058	PPP2R3B	X:294697-	10.2238	4.85315	-1.07494	-2.61676	5.00E-05	0.001069	yes
XLOC_058	ASMTL	X:1520661	40.3285	19.4291	-1.05358	-5.74612	5.00E-05	0.001069	yes
XLOC_058	DHRSX,ZBI	X:2137556	25.509	11.6279	-1.13342	-3.99079	5.00E-05	0.001069	yes
XLOC_059	ZMAT1	X:1011372	1.39601	0.620632	-1.1695	-2.87702	5.00E-05	0.001069	yes
XLOC_059	CAPN6	X:1104883	2.82851	0.053114	-5.7348	-4.38198	5.00E-05	0.001069	yes
XLOC_059	MBNL3	X:1313370	2.58528	0.571692	-2.17701	-1.92937	5.00E-05	0.001069	yes
XLOC_059	RPS4Y1	Y:2709526	283.567	52.8112	-2.42477	-14.7457	5.00E-05	0.001069	yes
XLOC_059	ZFY	Y:2803111	3.95018	1.01762	-1.95672	-3.39346	5.00E-05	0.001069	yes

XLOC_059	PRKY,RNU	Y:7142012	5.57146	1.28579	-2.11541	-1.96576	5.00E-05	0.001069	yes
XLOC_059	TTTY15	Y:1477426	8.0607	1.81169	-2.15357	-2.41547	5.00E-05	0.001069	yes
XLOC_059	USP9Y	Y:1481315	11.0278	2.06147	-2.4194	-6.88958	5.00E-05	0.001069	yes
XLOC_059	DDX3Y	Y:1501601	46.2892	8.80035	-2.39504	-7.12887	5.00E-05	0.001069	yes
XLOC_059	RP11-576C	Y:2172919	48.7509	8.82905	-2.4651	-8.58423	5.00E-05	0.001069	yes
XLOC_059	EIF1AY	Y:2273761	21.9494	4.28592	-2.35651	-10.4585	5.00E-05	0.001069	yes
XLOC_059	UTY	Y:1536025	5.28985	1.02523	-2.36729	-6.18782	5.00E-05	0.001069	yes
XLOC_060	TTTY14	Y:2103438	7.64033	0.69035	-3.46824	-3.42923	5.00E-05	0.001069	yes
XLOC_060	KDM5D	Y:2186575	20.1954	3.85019	-2.39102	-7.51271	5.00E-05	0.001069	yes
XLOC_009	CHST1	11:456704	0.39176	1.2006	1.61572	2.13007	0.0001	0.00197	yes
XLOC_011	MSRB3	12:656724	1.30025	3.30108	1.34415	1.51549	0.0001	0.00197	yes
XLOC_011	CPNE8	12:390406	3.74245	7.70012	1.0409	2.23211	0.0001	0.00197	yes
XLOC_013	STARD13	13:336772	0.48253	1.24809	1.37102	1.51877	0.0001	0.00197	yes
XLOC_013	KIAA0226L	13:469161	0.2173	0.945268	2.12105	0.85358	0.0001	0.00197	yes
XLOC_014	IFI27	14:945711	9.74416	3.69457	-1.39913	-1.99118	0.0001	0.00197	yes
XLOC_017	SLCO3A1	15:923969	4.52358	1.65326	-1.45216	-1.93364	0.0001	0.00197	yes
XLOC_022	RDM1	17:342450	1.32904	0.412572	-1.68767	-0.91065	0.0001	0.00197	yes
XLOC_024	CTD-2006C	19:120984	0.04001	1.16499	4.86374	0.37882	0.0001	0.00197	yes
XLOC_026	FAM83E	19:491040	1.56434	3.50796	1.16508	2.18186	0.0001	0.00197	yes
XLOC_029	LRP1B	2:1409889	0.80939	0.19807	-2.03082	-1.27274	0.0001	0.00197	yes
XLOC_030	RP11-366L	2:1827525	0.61207	0.285388	-1.10078	-2.68496	0.0001	0.00197	yes
XLOC_034	LINC00960	3:7572154	1.74246	0.70108	-1.31348	-2.72	0.0001	0.00197	yes
XLOC_039	LINC01021	5:2747239	2.37935	0.978973	-1.28123	-1.89737	0.0001	0.00197	yes
XLOC_039	IL31RA	5:5514720	0.37055	0.776135	1.06665	2.18357	0.0001	0.00197	yes
XLOC_041	RBM24	6:1728157	1.0564	2.43874	1.20697	1.9492	0.0001	0.00197	yes
XLOC_044	FAM221A	7:2371974	0.10921	1.16861	3.41959	1.05201	0.0001	0.00197	yes
XLOC_000	SAMD11	1:860259-	15.4788	4.29935	-1.8481	-1.83897	0.00015	0.002769	yes
XLOC_010	PRPH	12:496864	8.29927	18.8331	1.18222	2.66004	0.00015	0.002769	yes
XLOC_020	DNAH2	17:762067	0.79005	0.354245	-1.15719	-1.97906	0.00015	0.002769	yes
XLOC_025	DHDH	19:494369	2.05222	4.36038	1.08727	2.22508	0.00015	0.002769	yes
XLOC_025	C3	19:667771	1.35118	0.382876	-1.81927	-0.70984	0.00015	0.002769	yes
XLOC_036	GPR87	3:1508034	2.07474	4.90065	1.24004	2.65891	0.00015	0.002769	yes
XLOC_038	PRDM5	4:1216060	1.24416	2.84805	1.19481	1.82606	0.00015	0.002769	yes
XLOC_040	CTC-573N1	5:1798685	2.76063	1.0231	-1.43205	-2.56523	0.00015	0.002769	yes
XLOC_050	SYK	9:9356406	0.6759	1.53716	1.18539	1.9699	0.00015	0.002769	yes
XLOC_022	AC124789	17:365846	0.04689	1.17236	4.64411	0.44117	0.0002	0.003554	yes
XLOC_030	NGEF	2:2335620	2.8941	13.0206	2.16961	2.22427	0.0002	0.003554	yes
XLOC_034	NAALADL2	3:1741563	1.57955	0.432694	-1.8681	-0.87347	0.0002	0.003554	yes
XLOC_044	FAXC	6:9971904	0.95468	2.56198	1.42417	2.38523	0.0002	0.003554	yes
XLOC_051	SH2D3C	9:1305005	0.24553	0.705567	1.5229	0.80503	0.0002	0.003554	yes
XLOC_000	PADI3	1:1757559	0.30611	0.656242	1.10018	2.40106	0.00025	0.004292	yes
XLOC_033	ZNF860	3:3169938	0.53538	1.32248	1.3046	2.04877	0.00025	0.004292	yes
XLOC_043	GPSM3,NC	6:3215854	0.64173	1.49235	1.21756	1.81339	0.00025	0.004292	yes
XLOC_050	RP11-611D	9:1394406	1.92204	4.98403	1.37468	2.29149	0.00025	0.004292	yes
XLOC_059	TMSB15A	X:1017686	2.68451	0.768298	-1.80492	-2.53951	0.00025	0.004292	yes
XLOC_000	HPDL	1:4579254	1.49481	0.620529	-1.26839	-2.43712	0.0003	0.004992	yes
XLOC_000	ZRANB2-A	1:7154700	0.441	1.31906	1.58067	0.78331	0.0003	0.004992	yes
XLOC_001	LAMTOR5-	1:1109054	2.14053	6.827	1.67328	1.55812	0.0003	0.004992	yes
XLOC_018	IL32	16:311529	0.45765	1.04376	1.18947	0.42956	0.0003	0.004992	yes
XLOC_024	SCN1B	19:355215	1.44615	3.12733	1.11271	2.03448	0.0003	0.004992	yes

XLOC_030	COL6A3	2:2382326	0.55401	1.36285	1.29864	1.20042	0.0003	0.004992	yes
XLOC_034	RP11-15N	3:9989841	1.07203	0.51791	-1.04957	-2.27072	0.0003	0.004992	yes
XLOC_013	GAS6-AS2	13:114567	0.28934	0.916249	1.66297	2.66952	0.00035	0.0057	yes
XLOC_016	DLL4	15:412215	0.55654	1.13599	1.02939	2.03416	0.00035	0.0057	yes
XLOC_019	DPEP1	16:896797	0.21302	1.12308	2.39838	0.96018	0.00035	0.0057	yes
XLOC_042	HIST1H2A	6:2710083	0.37329	0.852466	1.19134	2.42445	0.00035	0.0057	yes
XLOC_046	ISPD	7:1613081	1.04761	0.429011	-1.28802	-2.11419	0.00035	0.0057	yes
XLOC_048	RP11-909N	8:1444949	1.683	4.57736	1.44348	2.57747	0.00035	0.0057	yes
XLOC_060	RP11-424	Y:2185382	0.98994	0.183378	-2.43252	-3.22943	0.0004	0.006391	yes
XLOC_010	GRASP	12:524007	1.20544	0.300726	-2.00304	-1.01027	0.00045	0.007086	yes
XLOC_004	RP11-101	1:1929813	4.93992	19.1593	1.95549	2.10991	0.0005	0.007675	yes
XLOC_037	ALB	4:7426283	0.71106	0.268481	-1.40514	-0.46512	0.0005	0.007675	yes
XLOC_059	ZNF736P9	Y:7936936	0.73641	0.128706	-2.51643	-2.47814	0.0005	0.007675	yes
XLOC_026	CTC-523E	19:352794	0.70657	0.229663	-1.62131	-1.66383	0.00055	0.0083	yes
XLOC_059	PNCK	X:1529351	0.38866	0.991513	1.35113	0.45985	0.00055	0.0083	yes
XLOC_002	FAM46B	1:2733151	0.28809	0.950191	1.7217	2.91895	0.0006	0.008922	yes
XLOC_015	CDKL1	14:507042	2.98495	7.82039	1.38954	2.12918	0.0006	0.008922	yes
XLOC_034	TF	3:1333808	3.3289	1.60663	-1.051	-1.34474	0.0006	0.008922	yes
XLOC_006	MAT1A	10:820315	2.02579	0.990045	-1.03292	-1.61282	0.00065	0.009508	yes
XLOC_026	NOVA2	19:464369	0.31721	0.732039	1.20648	1.47073	0.00065	0.009508	yes
XLOC_030	ICA1L	2:2036406	0.77669	1.6048	1.04698	0.77337	0.00065	0.009508	yes
XLOC_046	SPDYE6	7:1019861	0.5587	1.475	1.40059	2.24416	0.00065	0.009508	yes
XLOC_008	TMPRSS4	11:117886	1.92173	0.76086	-1.3367	-1.08122	0.0007	0.010084	yes
XLOC_022	KRTAP2-3	17:392154	1.85292	4.24937	1.19745	2.33956	0.0007	0.010084	yes
XLOC_022	AC003665	17:459735	1.49956	3.58005	1.25544	1.95687	0.0007	0.010084	yes
XLOC_023	ANKRD20A	18:141790	8.17932	3.7506	-1.12486	-1.8295	0.0007	0.010084	yes
XLOC_029	LIMS2	2:1283959	0.71509	1.51584	1.08391	1.20658	0.0007	0.010084	yes
XLOC_004	CAPN8	1:2237113	2.10875	1.0195	-1.04853	-1.30886	0.00075	0.010764	yes
XLOC_037	FGF2	4:1237478	0.68153	2.68786	1.9796	1.15009	0.0008	0.011374	yes
XLOC_051	RP11-92C4	9:1015699	0.15803	1.18089	2.9016	0.56331	0.0008	0.011374	yes
XLOC_005	IFIT1	10:909733	0.79287	2.31407	1.54528	2.17753	0.00085	0.011929	yes
XLOC_027	DYSF	2:7168085	1.01572	2.04702	1.01102	1.38707	0.00085	0.011929	yes
XLOC_041	ZNF608	5:1239726	0.64372	1.79776	1.4817	1.51396	0.00085	0.011929	yes
XLOC_002	PPFIA4	1:2029956	0.58109	1.41762	1.28664	1.11479	0.00095	0.013103	yes
XLOC_010	ROBO4	11:124753	0.18369	0.906446	2.30299	0.96359	0.001	0.013608	yes
XLOC_009	CHRM4	11:464066	0.41959	1.54682	1.88225	2.63677	0.0011	0.014616	yes
XLOC_010	CD4	12:689602	0.40425	1.16902	1.532	0.98899	0.0011	0.014616	yes
XLOC_013	LINC0046	13:107028	2.46793	5.61291	1.18545	2.20657	0.0011	0.014616	yes
XLOC_034	C3orf55	3:1572610	0.94873	0.120685	-2.97474	-1.13485	0.0011	0.014616	yes
XLOC_012	TSPAN19	12:854080	0.32439	1.07069	1.72275	0.81067	0.00115	0.015148	yes
XLOC_024	CATSPERG	19:388264	1.19399	2.93407	1.29711	1.58008	0.0012	0.015685	yes
XLOC_029	CACNB4	2:1526892	0.30457	0.746489	1.29333	1.3086	0.0012	0.015685	yes
XLOC_008	RP11-169	11:722817	0.21947	0.598487	1.44729	2.2583	0.00125	0.016158	yes
XLOC_026	KHK	2:2730961	1.213	2.49459	1.04022	1.57792	0.00125	0.016158	yes
XLOC_049	SMARCA2	9:2015341	1.45779	3.00716	1.04462	1.51442	0.00125	0.016158	yes
XLOC_051	ABO,RP11	9:1361257	0.35105	0.826413	1.2352	1.87484	0.00125	0.016158	yes
XLOC_017	CYP11A1	15:746300	0.90746	0.394619	-1.20138	-0.93769	0.0013	0.016677	yes
XLOC_058	TMSB15B	X:1031720	0.90346	0.208638	-2.11447	-1.31304	0.0013	0.016677	yes
XLOC_003	RP11-640	1:1442759	1.19214	2.67808	1.16764	1.05124	0.0014	0.017662	yes
XLOC_008	SLC22A20	11:649813	0.21043	0.902071	2.09993	1.20421	0.00145	0.018083	yes

XLOC_041	ID4	6:1953517	1.00987	0.422817	-1.25607	-2.14451	0.00145	0.018083	yes
XLOC_006	DUSP13	10:768541	0.21408	0.846738	1.98376	0.48113	0.0015	0.018615	yes
XLOC_015	LTBP2	14:749648	0.71362	1.44424	1.01708	1.22846	0.0015	0.018615	yes
XLOC_007	COL17A1	10:105791	3.44811	1.42532	-1.27452	-1.3516	0.00155	0.019095	yes
XLOC_031	AP000330	21:361180	0.43589	0.933979	1.09944	1.70279	0.00155	0.019095	yes
XLOC_000	FHAD1	1:1557376	1.61293	3.25671	1.01373	1.26184	0.00165	0.020036	yes
XLOC_037	SLC2A9	4:9772776	3.10669	1.43889	-1.11043	-1.31635	0.00165	0.020036	yes
XLOC_042	RUNX2	6:4477705	0.21898	0.65616	1.58326	0.77651	0.00165	0.020036	yes
XLOC_028	CCL20	2:2286785	2.34938	1.04991	-1.16201	-1.72518	0.00175	0.021015	yes
XLOC_057	ARSD-AS1	X:2822010	8.9371	4.3827	-1.02799	-1.88406	0.00175	0.021015	yes
XLOC_037	PRDM8	4:8110443	0.12759	0.677815	2.40939	0.63533	0.0018	0.021514	yes
XLOC_025	ZNF530	19:581112	1.42524	0.592001	-1.26753	-1.65591	0.00185	0.022008	yes
XLOC_003	RP11-315I	1:1454759	0.35007	1.01652	1.5379	0.31388	0.00195	0.023	yes
XLOC_048	KB-1732A1	8:1038177	1.59503	0.529194	-1.59172	-1.66598	0.002	0.02348	yes
XLOC_000	GRHL3	1:2463404	1.30552	0.435784	-1.58294	-1.08302	0.00205	0.023957	yes
XLOC_018	KREMEN2	16:301394	2.8715	6.1164	1.09088	1.95014	0.00205	0.023957	yes
XLOC_033	GGT5	22:246156	1.13102	0.346003	-1.70877	-1.32794	0.00205	0.023957	yes
XLOC_007	RP11-326C	11:287304	1.16724	2.98693	1.35557	1.49583	0.0021	0.024317	yes
XLOC_048	LINC00589	8:2953143	1.24098	0.178189	-2.8	-2.69045	0.0021	0.024317	yes
XLOC_048	ANGPT2	8:6261071	0.11093	0.634436	2.51587	1.74809	0.0022	0.025226	yes
XLOC_028	AC092594	2:1916772	1.51169	0.711557	-1.08711	-1.47156	0.0023	0.026097	yes
XLOC_038	DDIT4L	4:1008692	8.03771	2.36035	-1.76778	-1.92278	0.0023	0.026097	yes
XLOC_043	RP11-420C	6:2854895	0.4959	1.09451	1.14217	1.66489	0.0023	0.026097	yes
XLOC_043	PLA2G7	6:4662067	0.38941	1.17382	1.59184	1.71726	0.00235	0.026526	yes
XLOC_002	RHOU	1:2288708	4.60837	1.85968	-1.3092	-1.49542	0.00245	0.027512	yes
XLOC_021	ASGR1	17:707674	0.27551	0.866245	1.65269	1.07603	0.00245	0.027512	yes
XLOC_030	TUBA4A	2:2200944	4.62228	9.44924	1.0316	1.67123	0.00245	0.027512	yes
XLOC_036	S100P	4:6694795	37.7571	16.4503	-1.19864	-1.81533	0.00255	0.028467	yes
XLOC_034	TMED10P2	3:1282568	2.56631	0.719695	-1.83424	-2.28546	0.00265	0.029283	yes
XLOC_036	SPTSSB	3:1610625	0.3141	1.0331	1.71767	1.76009	0.00265	0.029283	yes
XLOC_000	ST6GALNA	1:7654040	0.93938	0.469528	-1.0005	-1.49754	0.0028	0.030651	yes
XLOC_006	RBM20	10:112404	0.49179	0.994657	1.01617	1.21055	0.0028	0.030651	yes
XLOC_006	C10orf10	10:453064	0.37958	1.97146	2.37679	2.33316	0.00295	0.031903	yes
XLOC_026	CA11	19:491411	6.51742	14.8524	1.18832	1.78485	0.00315	0.033425	yes
XLOC_011	NAV3	12:782246	0.34038	0.859412	1.33619	0.65532	0.0032	0.033721	yes
XLOC_035	RP11-379E	3:1255460	0.69886	1.56152	1.15987	1.04232	0.0032	0.033721	yes
XLOC_034	RP11-260C	3:8203528	2.98731	1.33475	-1.16227	-1.67689	0.0033	0.034559	yes
XLOC_027	ATOH8	2:8597846	0.78078	0.279915	-1.47993	-1.31167	0.0034	0.035291	yes
XLOC_018	NR2F2-AS1	15:966705	0.37152	1.65743	2.15745	0.94378	0.00345	0.035664	yes
XLOC_021	ALOX12B	17:797595	1.9679	0.781409	-1.33251	-1.57988	0.0035	0.036083	yes
XLOC_050	LCN12	9:1398440	0.66459	1.40226	1.07721	0.71162	0.00355	0.03645	yes
XLOC_004	RPL21P28	1:2122089	13.265	2.7682	-2.26061	-2.01781	0.00365	0.037326	yes
XLOC_020	AC040160	16:672410	0.27823	0.767536	1.46395	0.78169	0.00395	0.039701	yes
XLOC_001	TRIM46	1:1551418	0.60621	1.85555	1.61395	1.70041	0.0041	0.040858	yes
XLOC_026	LYPD5	19:443000	0.42435	1.13254	1.41624	0.95418	0.00415	0.041248	yes
XLOC_041	FOXQ1	6:1312674	1.18345	0.464832	-1.34822	-2.07924	0.00455	0.044157	yes
XLOC_000	MAN1C1	1:2594395	0.37759	0.762042	1.01303	1.22131	0.0046	0.044614	yes
XLOC_012	TRHDE-AS	12:723326	0.03866	0.599204	3.95424	0.67285	0.00465	0.04487	yes
XLOC_022	GPR179	17:364814	0.26737	0.6791	1.34481	1.01974	0.00475	0.045518	yes
XLOC_025	SLC7A9	19:333214	0.88068	0.254295	-1.79211	-1.40057	0.00475	0.045518	yes

XLOC_023	RAB27B	18:523850	0.36721	0.903752	1.29932	0.92289	0.00485	0.046244	yes
XLOC_010	CNTN1	12:410862	0.39426	1.0073	1.35328	0.66311	0.0049	0.046516	yes
XLOC_057	MAGEB1,M	X:30260005	0	0.857728	inf	#NAME?	5.00E-05	0.001069	yes
XLOC_051	IGKV1OR-2	9:69777709	0	0.785329	inf	#NAME?	0.00115	0.015148	yes
XLOC_034	RP11-329B	3:18445970	0	0.654688	inf	#NAME?	0.00145	0.018083	yes
XLOC_014	TRAV18	14:224713	0.75955	0	#NAME?	#NAME?	0.00135	0.017145	yes
XLOC_048	KB-1410C5	8:1022203	1.29773	0	#NAME?	#NAME?	0.00135	0.017145	yes
