# Supplementary material for: A 16q22.1 variant confers susceptibility to colorectal cancer as a distal regulator of ZFP90
Source: Oncogene. 2019 Oct 22;39(6):1347–60. doi: 10.1038/s41388-019-1055-4 (PMC7002302; doi:10.1038/s41388-019-1055-4)
Supplement: Supplementary file 10 — Table S4 [file 41388_2019_1055_MOESM10_ESM.pdf]

Table S4: Screen of ZFP90 target genes

ZFP90 ChIP-seq	RNA-seq (WT vs ZFP90-KO)	Overlap (6)
BMP4	BMP4	BMP4
GATA2	FXYD4	FXYD4
PDE4D	GATA2	GATA2
SERPING1	CASP10	CASP10
FXYD4	SERPING1	SERPING1
CASP10	PDE4D	PDE4D
MISP	CAPN6	
ERBB2	EPS8L3	
ST8SIA6	COX6B2,CTD-2105E13.6,FAM71E2	
IFNG	TTTY14	
COL9A3	AL162759.1	
ALDH3B2	KRT13	
FCGR3B	ZNF701,ZNF808	
FADS3	RP11-435B5.5	
LOC645513	HPD	
RPAP3	ANXA10	
ZNF705G	SEMA3A	
NR5A2	C3orf55	
MIR3686	MYRIP	
ADAMTSL5	LINC00589	
FGFR2	STK32A	
LOC388882	ARHGDIB	
NR1H4	EPGN	
PCDHB17P	LY6D	
STK31	ZNF736P9Y	
MLH3	RP11-576C2.1,TXLNG2P	
MIR5580	KRT20	
DICER1	RP11-424G14.1	
BRPF1	RPS4Y1	
STAT1	USP9Y	
LOC645752	P2RX7	
GUCA2A	SULT1C2	
LOC100507346	DDX3Y	
FAM174A	KDM5D	
RTTN	UTY	
RPS6KC1	EIF1AY	
MIEN1	RPL21P28	
PCDHB8	TBX3	
TRIM11	TNFRSF9	
IL21R-AS1	MBNL3	
C1orf216	TTTY15	
KRTAP10-5	C11orf86	
MOGS	PRKY,RNU6-941P	
GATA2-AS1	TMSB15B	

NRAS	LRP1B	
SLX4	GRASP	
UNC119	CYP4F35P	
MIR3138	ZFY	
LIPH	NAALADL2	
RNASEL	NLRP12	
PTENP1	SAMD11	
LOC100240735	TMED10P2	
ICAM4	C3	
TUBB3	TMSB15A	
PTENP1-AS	SLC7A9	
CFB	ALOX5	
C8B	DDIT4L	
RNASEH2C	METTL7A	
C1R	TRIM29	
NFKBIA	ABCC2	
ATF1	SOX4	
LCN8	PRF1	
PDHB	GGT5	
C11orf74	RDM1	
HTR1A	TUBAL3	
MIR548F2	PRKAA2	
BCL6	STON2	
CYP27B1	CTC-523E23.4	
PSG7	FRMD3	
LOC101927142	CYP4F12	
PSG10P	TNFSF4	
ZBTB7B	KB-1732A1.1	
LOC101927055	SGPP2	
IL2RG	GRHL3	
POU2F1	PBX1	
CD8A	MAN1A1	
GIMAP1-GIMAP5	FGFBP1	
GIMAP1	SLIT2	
MIR4728	KRT16	
PCAT2	PELI2	
TPRN	ATOH8	
MAGEL2	ID3	
EEF1G	SIK1	
ACP5	CPEB2	
TPM2	SLCO3A1	
RPS20	C9orf3	
NDNL2	CTC-573N18.1	
HPSE2	ABCA12	
TP53	ALB	
PI4K2B	IFI27	

ITPA	SPRR2D	
KRT9	EPHA4	
MASP1	SESN3	
JAZF1	FOXQ1	
PYGL	TMPRSS4	
PTCH1	ALOX12B	
OR1E1	SLC2A1	
NBPF13P	LINC00960	
KMT2A	RHOU	
SNORD54	AC068138.1	
HTR2A	ISPD	
LOC100129216	LINC01021	
MSMB	COL17A1	
MNAT1	CSF2RA	
JAZF1-AS1	HPDL	
HIST1H2BF	ZNF530	
WDR54	ID4	
LOC642943	HTR7	
FOXP2	DLX2	
GALR3	PLCXD1	
OR56B1	AC003005.4,ZNF419,ZNF773	
MET	CYP11A1	
FASLG	SDR16C5	
SULT1C4	S100P	
POTED	H19	
SUGT1	RN7SL2	
HIST1H3D	ZMAT1	
HIST1H2AD	DPYSL3	
KRTAP5-9	CASC5	
LACE1	RP11-260O18.1	
NOTCH4	CCL20	
CFAP20	SVIL	
NKX2-3	DNAH2	
TRAF6	NLRP11	
TRIM31-AS1	FOXO1	
LRG1	FABP6	
MVB12A	DHRSX,ZBED1	
FAM181A	ANKRD20A5P,RP11-757O6.1	
CHRAC1	CDCA5	
HAX1	TPBG	
PRNCR1	SLC2A9	
MVK	KRT23	
GAGE10	RP11-43F13.3	
SPTAN1	SEMA3C	
MMAB	RP11-366L5.1	
CCDC159	IQGAP3	

TECR	AC092594.1	
MIR639	PPP2R3B	
FAM47C	NLRP2	
CDYL	PPAP2B	
PCDHB6	IBTK	
MSL3P1	NEBL	
PCDHB7	ASMTL	
TMEM205	TF	
POM121L4P	RP11-15N24.4	
PCDHA12	CAPN8	
C2-AS1	MAT1A	
LOC728392	ARSD-AS1	
LINC01475	IL8	
LOC101928673	RRM2	
SNAP91	RP11-54O7.3	
SNORA38	PALLD	
ZNF718	RGS16	
CXCR5	FOSL2	
EGFR-AS1	DFNA5	
SLC14A1	SYTL2	
MC1R	ST6GALNAC3	
FAM66B	RP11-111G23.1	
MIOX	FKBP5	
ATP4B	FGD6	
C11orf91	DYSF	
CLP1	ADAMTS10	
CXorf65	MAN1C1	
KRTAP10-4	FHAD1	
WBP1	SLC7A2	
MIR378G	STOM	
SLC25A51P1	RBM20	
TRY2P	LTBP2	
HEATR3	BZW1	
MAATS1	ZEB1-AS1	
TTN-AS1	LIMA1	
RNF31	ZBTB18	
C2orf81	C16orf52	
RTEL1	DLL4	
RTEL1-TNFRSF6B	LINC00896	
AKAP8L	TUBA4A	
PKD1L1	DRD1	
TCN1	SECTM1	
CEP131	KHK	
DDRGK1	CPNE8	
NBPF8	SMARCA2	
NBPF9	ICA1L	

ZNF484	KLF15	
WDR47	ETS1	
PSME2	IL31RA	
PRRC2A	PRKAR2B	
CCR5	TMEM55A	
	ADM	
	CDC73	
	LCN12	
	TEF	
	NT5E	
	LIMS2	
	MLPH	
	DHDH	
	GRB7	
	KREMEN2	
	AP000330.8	
	PADI3	
	CDC42EP2	
	RP11-290F20.1	
	PPP4R4	
	GATA6	
	SLC37A2	
	CCDC68	
	SCN1B	
	KCNK13	
	OSBPL10	
	RP11-986E7.7,SERPINA3,SERPINA4,SERPINA5	
	LAMA3	
	DGKH	
	RP11-420G6.4	
	PDGFC	
	CYP24A1	
	RP11-379B18.5	
	NES	
	FAM83E	
	CHST7	
	RP11-640M9.1	
	MT1E	
	MT2A	
	PDE2A	
	CALB2	
	C1orf116	
	PRPH	
	SYK	
	LINC00460	
	MB	

	CA11	
	IL32	
	HIST1H2AG	
	PRDM5	
	KRTAP2-3	
	ELK3	
	NOVA2	
	RBM24	
	H3F3AP4	
	RNF128	
	PXDN	
	GPSM3,NOTCH4	
	RP11-350N15.6	
	FEZF1-AS1	
	ABO,RP11-430N14.4	
	GPR87	
	PAPL	
	STK39	
	LEMD1	
	HOXB3,HOXB4,HOXB6	
	SKAP1	
	ARL4C	
	PER2	
	AC003665.1	
	MRC2	
	CASC10	
	COL12A1	
	PPP1R14C	
	C6orf1	
	LCN2	
	PPFIA4	
	CACNB4	
	CATSPERG	
	RHOBTB3	
	COL6A3	
	RAB27B	
	ZNF860	
	UNC13A	
	TACSTD2	
	FLNC	
	FHL1	
	BVES	
	TNIK	
	MBOAT2	
	OLFML2A	
	LYRM7	

	NAV3	
	MSRB3	
	GPR179	
	PNCK	
	CNTN1	
	RP11-326C3.2	
	GLIS3	
	ZNF467	
	UCHL5	
	STARD13	
	RP11-611D20.2	
	SYNE3	
	ENKD1	
	LINC00341	
	CDKL1	
	TMEM200A	
	SPDYE6	
	LMOD1	
	AFAP1L1	
	LYPD5	
	LETM2	
	FAXC	
	SMURF2	
	RP11-66B24.4	
	POPDC3	
	RP11-909N17.2	
	RP11-169D4.1	
	ALDH1A3	
	MT1X	
	AC040160.1,LRRC29	
	RP11-13A1.1	
	ZNF608	
	PDGFB	
	CCDC136	
	NRP1	
	SKAP2	
	SH2D3C	
	TIAM1	
	CD4	
	RP11-315I20.1	
	MGLL	
	SUN3	
	IFIT1	
	CDK6	
	ARHGAP29	
	HOXA1	

	ZRANB2-AS2	
	RUNX2	
	MRVI1	
	PLA2G7	
	SERPINA1	
	TRIM46	
	CHST1	
	PLAU	
	ASGR1	
	SSPO	
	GAS6-AS2	
	PGM5P2,RP11-87H9.3	
	LAMTOR5-AS1	
	RP11-381O7.3	
	HOTAIRM1	
	ACHE	
	CTGF	
	PRKCDBP	
	SPTSSB	
	FAM46B	
	TSPAN19	
	PRSS8	
	INPP4B	
	GLRX2	
	SERPINE1	
	ABCC6	
	HOXA3,HOXA4,HOXA6	
	RAMP1	
	PRAME	
	F3	
	VGF	
	TROVE2	
	CHRM4	
	RP11-909N17.3	
	STRA6	
	COL13A1	
	RP11-101E13.5	
	FGF2	
	CYTH4	
	DUSP13	
	AC005152.3,RP11-84E24.2,SOX9-AS1	
	RP11-230G5.2	
	SLC22A20	
	KIAA0226L	
	TRNP1	
	GALNT5	

	NR2F2-AS1	
	TCEA3	
	NFIB	
	NGEF	
	GPR110	
	GLI2	
	ROBO4	
	HOXA-AS2	
	MDGA1	
	C10orf10	
	ITGB8	
	DCN	
	DPEP1	
	PRDM8	
	LCNL1,PTGDS	
	SLC1A3	
	B3GALT5	
	ALDH1L2	
	ANGPT2	
	LAMA4	
	DACT1	
	SQLE	
	NAMPTL	
	RP11-92C4.3	
	RP3-323P13.2	
	TCL1B	
	NRG1	
	C1orf106	
	FAM221A	
	TPH2,TRHDE	
	DSC3	
	TRHDE-AS1	
	AC124789.1	
	CTD-2006C1.2	
	MAGEB1,MAGEB4	
	IGKV1OR-2	
	RP11-329B9.3	
	TRAV18	
	KB-1410C5.5	
