# Supplementary material for: A 16q22.1 variant confers susceptibility to colorectal cancer as a distal regulator of ZFP90
Source: Oncogene. 2019 Oct 22;39(6):1347–60. doi: 10.1038/s41388-019-1055-4 (PMC7002302; doi:10.1038/s41388-019-1055-4)
Supplement: Supplementary file 11 — Table S5 [file 41388_2019_1055_MOESM11_ESM.pdf]

**Table S5: Sequences of mRNA siRNAs, primers of genes and gDNA, sgRNA Related to STAR Methods**		
**Name**	**Sense (5'-3')**	**Antisense (5'-3')**
ZFP90 siRNA	GCAGCAGGAUGUAUCAGAATT	UUCUGAUACAUCCUGCUGCTT
NFATC1 siRNA	GGUCAUUUUCGUGGAGAAATT	UUUCUCCACGAAAAUGACCTT
NFATC2 siRNA	CCAUUAAACAGGAGCAGAATT	UUCUGCUCCUGUUUAAUGGTT
NFATC3 siRNA	GGUUGUGACUGGAUCUAAUTT	AUUAGAUCCAGUCACAACCTT
NFAT5 siRNA	GUACCUCGUAAAUCACGAATT	UUCGUGAUUUACGAGGUACTT
PRDM1 siRNA	GGAAAGGACCUCUACCGUUTT	AACGGUAGAGGUCCUUUCCTT
BMP4 siRNA	GCCAGGAAGAAGAAUAAGATT	UCUUAUUCUUCUUCCUGGCTT
**sgRNA**	**Top (5'-3')**	**Bottom (5'-3')**
HCT116 *ZFP90*-KO	AGAAGAGCCATGGATATCAG	CTGATATCCATGGCTCTTCT
DLD1 *ZFP90*-KO	TCCTCTGAGCAGGGTCGACA	TGTCGACCCTGCTCAGAGGA
△S3-5 sgRNA6	CCTCAGTATATCTTTAATCCCTT	AAGGGATTAAAGATATACTGAGG
△S3-5 sgRNA11	CCATGGAGCATTTCTGCAAGAG C	GCTCTTGCAGAAATGCTCCATGG
△S2 sgRNA3	CCACCCTGGATTGGCTTCCAACC	GGTTGGAAGCCAATCCAGGGTGG
△S2 sgRNA8	CCCGCAGCACTCATTTTAACCCC	GGGGTTAAAATGAGTGCTGCGGG
△RR sgRNA1	ATCTCCTTGTGGTGGTAGCA	TGCTACCACCACAAGGAGAT
△RR sgRNA9	ACACGTAATGACTGTCATAG	CTATGACAGTCATTACGTGT
SNP-rs7198799_CT to TT sgRNA9	GCATGGTGTTGGTTATGTGA	TCACATAACCAACACCATGC
SNP-rs7199991_TG to GG sgRNA3	CTGTCAATGATGTGTCTATC	GATAGACACATCATTGACAG
SNP-rs7198799_CT to CC sgRNA9	GCATGGTGTTGGTTATGTGA	TCACATAACCAACACCATGC
Zfp90 Global Knockout Mice sgRNA1	TTCGACAGAATCGTACACGC	GCGTGTACGATTCTGTCGAA
Zfp90 Global Knockout Mice sgRNA2	CCGCTACAATAGTGAGACCT	AGGTCTCACTATTGTAGCGG
Zfp90 Conditioned Knockout Mice sgRNA1	GCTATCTAATAGGTTACTGT	ACAGTAACCTATTAGATAGC
Zfp90 Conditioned Knockout Mice sgRNA2	CCGCCCCAAGTCAATTTTAC	GTAAAATTGACTTGGGGCGG
**Primers of 3C-seq inverse PCR**	**Forward**	**Reverse**
ZFP90 TSS	ACGCCACCACACCCTGCTAA	GGTGCAAAAGCTGTCAGATGG
rs9929218(RR)	CCATGCCTGGCTTGTTT	TACCTGGACTGTTCCCATTT
rs7198799(RL)	AACAATGCCTTAGTGAATAACC	AAGCTCTTTATGCTCTGCA
**Primers of 3C-qPCR**	**Forward**	**Reverse**
**SNP region（RL+RR）（ZFP90 TSS bait）**		
P1	CAGGGACACTGCCTTTCTTAT	TTCCAGGCTGAAGAAACTGAA
P2	GGCTGACTTCAGACTTGTTACT	AACTGCAGGTGCATAAGGG
P3 (rs7198799, RL)	GCTAGCACAAACAATGCCTTAG	TTCCAGGCTGAAGAAACTGAA
P4 (rs7198799 RR)	CCCATCCCACATTGCTGTTA	TCCAGGCTGAAGAAACTGAAG
P5	CAGTGAAGACAGAGACGATACA TAA	TTCCAGGCTGAAGAAACTGAA
**ZFP90 TSS (rs7198799, RL bait)**		
P1	CCTGGGTGAAAGAGTGAAACT	ACATTGGTGCCTCTAAGCC
P2	GGACTCAAAGAGTGAGCAGTAG	ACATTGGTGCCTCTAAGCC

P3	CTGCTCTGCTCACTCTCTTATC	ACATTGGTGCCTCTAAGCC
P4 (ZFP90 TSS)	GTAACTAGATGGCTACGGTTCTG	ACATTGGTGCCTCTAAGCC
P5	GTGGCAGAAGCTTGTGTTTG	GTTCAGAGAGAGAAAGCCACAG
P6	AAGACCAGCCTCCCTTCT	GGTTCAGAGAGAGAAAGCCACAG
**ZFP90 TSS (rs9929218, RR bait)**		
P1	CCTGGGTGAAAGAGTGAAACT	AAATGGGAACAGTCCAGGTAAA
P2	GAGTGAGCAGTAGCAAGGTTTA T	CATTTCTGCAAGAGCAGCAAC
P3	CTCACTCTCTTATCCCTCACTCT	AACAGTCCAGGTAAATGGCC
P4 (ZFP90 TSS)	ACGGTTCTGAAATGGAGATGG	AAGAGCAGCAACCAGGAAA
P5	TGTTGTTACTGGGCCAAAGAG	CATTTCTGCAAGAGCAGCAAC
P6	CCTCCCTTCTCAAGCTTTCC	AAGAGCAGCAACCAGGAAA
**Primers of PCR**		
△S3-5		
HR (homologous recombination) allele PCR screening		
CL-WFZ-003-L-GT-F/PU△TK-GT-F	AGCACTCCCTCCTTATCCTTCCTC C	CTCGACTGTGCCTTCTAGTTGCCAG
Neo-GT-R/CL-WFZ-003-R-GT-R	CAGAGGCCACTTGTGTAGCG	ACAGGAAAGCCGTTGTGGAATTCAG
FS(Frameshift) allele PCR screening		
CL-WFZ-003-FS-F1/CL-WFZ-003-FS-R1	CTTCATTCTCCACCCTCCTCCACA C	TCCCTTCTCAGGCCATCTGGAAACA
Del (Deletion) allele PCR screening		
CL-WFZ-003-Del-F/CL-WFZ-003-Del-R	CTTCATTCTCCACCCTCCTCCACA C	GAGTGACCCACAGGTGCCAGAATC
△S2		
HR (homologous recombination) allele PCR screening		
CL-WXH-001-L-GT-F/PU△TK-GT-F	GTCAGAAGGCAGGAAGTTCTCT GGG	GTCGACTGTGCCTTCTAGTTGCCAG
SV40pro-R/CL-WXH-001-R-GT-R	GAATAGCTCAGAGGCAGAGG	CCTGTCACAGTTGGTGGGAACATTT
FS(Frameshift) allele PCR screening		
CL-WFZ-001-FS-F1/CL-WFZ-001-FS-R1	CATCCCCAGCTGAAACCCTCTCA T	GTAGATCACTTGAAATCAGCCTGGCC
Del (Deletion) allele PCR screening		
CL-WFZ-001-Del-F/CL-WFZ-001-Del-R	CATCCCCAGCTGAAACCCTCTCA T	CAGGTGTAGTGGCATGCTCCTGT

SNP-rs7198799 CT to TT		
CL-LS-002-L-GT-F	GTGTGCTCTTTAAAGACAGATTCTTCCC	
CL-LS-002-△TK-R		GCCCACGCTACTGCGGGTTTATATAG
CL-LS-002-△TK-F	CGGCATAAGGCATGCCCATTGTTATC	
CL-LS-002-R-GT-R		GCAAGTGCAAAGCAGTGCTATCTTTTG
SNP-rs7199991 TG to GG		
CL-LJL-015-L-GT-F	GGTCATCAGATTGCAACCCTGAGAATG	
Puro-GT-F		GCAACAGATGGAAGGCCTCCTGGCG
Puro-GT-R(LJL)	TAAGTCATCGGCTCGGGTACGTAGA	
CL-LJL-015-R-GT-R		CCCAGGAGTTTGAGTCTGCAGTGAG
ZFP90$^{+/-}$ mice		
P1/P2	TGGTGGTGTTCGCCTTTAGTCTCA	GCCCTGCTCAAGTCGGAATCAAC
P3/P4	GGGGATAGGAAGGCTTGGTGATG	GCCTCGCCCTGTGTTTTGTCTT
ZFP90$^{fl/+,Villin\text{-}cre/+}$ mice		
EGE-WNN-007-L-GT-F/cKO-3'-DO-R	AGGTGCCTGGTGTTTCCCTTAGTTT	GACGCCTAGATTGTGCTACTCTCAGCT
cKO-5'-DO-F/EGE-WNN-007-R-GT-R	CGTGCTAGATCGACTGCTAGAGTGAC	ATGAGGGAGAAGAGGTGGGTAGCTG
EGE-WNN-007-5'loxP-F/EGE-WNN-007-5'loxP-R	ACTCTGCTCTCGTCCCAGATGTTGA	AGAGTGCCTGTCACATCCAGAGAGT
EGE-WNN-007-3'loxP-F/EGE-WNN-007-3'loxP-R	TCAACCGATGTATCATTCCATTGGC	GAGTTAAGATGGGGGTTGAAACAGC
PVillin-cre-P1/PVillin-cre-P2	AGCGATGGATTTCCGTCTCTGG	AGCTTGCATGATCTCCGGTATTGAA
**Primers of Real-time PCR**	**Sense (5'-3')**	**Antisense (5'-3')**
ZFP90	GAGAGGCAAACCTTACCAAAGC	CCTGCTCACAATCATAGGGGT
CDH1	AGAACGCATTGCCACATACACTC	CATTCTGATCGGTTACCGTGATC
NFATC2	GAGGGGCTGTCAAAGCTCC	ACAGTTTTCCCCGTGATTCGG
BMP4	CGTAGCCCTAAGCATCACTCACA	GCGCCGGCAGTTCTTATTCT
FXYD4	TTGCCAATAAAGACGATCCCTTC	AGAGCCTGGAGTGATGAGTGG
GATA2	ACTGACGGAGAGCATGAAGAT	CCGGCACATAGGAGGGGTA

CASP10	AGAAACCTGCTCTACGAACTGT	GGGAAGCGAGTCTTTCAGAAG
ST8SIA6	ATGAGTTACGAGGTGGAAAGCA	CCGACCACTGCACACTGATTA
SERPING1	CTGGCTGGGGATAGAGCCT	GAGATAACTGTTGTTGCGACCT
PDE4D	ACGGACCGGATAATGGAGGAG	ATTTTTCCACGGAAGCATTGTG
GAPDH	GCATTGCCCTCAACGACCAC	CCACCACCCTGTTGCTGTAG
F. nucleatum	CAACCATTACTTTAACTCTACCATGTTCA	GTTGACTTTACAGAAGGAGATTATGTAAAAATC
16S rRNA	GGTGAATACGTTCCCGG	TACGGCTACCTTGTTACGACTT
**Primers of ChIP-qPCR**	**Sense（5'-3'）**	**Antisense（5'-3'）**
BMP4-1	CGGCAGCATCTTCGATTAGT	AGTCGCACGCAGCAAATA
BMP4-2	AGGCAACCCGAGTTCTTG	CCCAAAGCCCACTCCAC
GATA2-1	ACGTGTCCCAAGCTTTCTG	GCACACCCTGTGCATCC
GATA2-2	CCCGCAAAGTGATGTCGAA	GTGGCGGCAGGCAATAG
SERPING1-1	TGGTTGTGTAAAGCTGAGAACT	CTGACCGCTTGGTCTTCTTT
SERPING1-2	CCCGACATTTCACTGCTCTAAG	GGAAATTGCTCACCTCCGTTAT
PDE4D-1	CCCAAAGGAAGGGAAGAAGAA	TCCTTCCACCGAGGCTAT
PDE4D-2	ACAGGTCTGACTCCTGACT	CACCGGAACCCTGATCC
rs7198799 region	CCCTTCCCTCTCCCATCA	GTTCACATAACCAACACCATGC
