# Supplementary material for: A 16q22.1 variant confers susceptibility to colorectal cancer as a distal regulator of ZFP90
Source: Oncogene. 2019 Oct 22;39(6):1347–60. doi: 10.1038/s41388-019-1055-4 (PMC7002302; doi:10.1038/s41388-019-1055-4)
Supplement: Supplementary file 12 — Table S6 [file 41388_2019_1055_MOESM12_ESM.pdf]

Table S6: Supplemeantary materials

REAGENTS or RESOURCES	SOURCE	IDENTIFIER
Antibodies		
Anti-GAPDH	Abcam	Cat#ab9482
Anti-ZFP90	Sigma-Aldrich	Cat#HPA029017
Anti-ZFP90	Sigma-Aldrich	Cat#SAB2103688
Anti-E-Cadherin (CDH1)	Cell Signaling Technology	Cat#3195
Anti-Ki67	Cell Signaling Technology	Cat#12202
Anti-IgG	Cell Signaling Technology	Cat#2729
Anti-BMP4	Abcam	Cat#ab124715
Anti-NFATC2	Abcam	Cat#ab2722
Biological Samples		
Snap-frozen colorectal cancer tissues and paired normal colorectal mucosa	Renji Hospital affiliated to Shanghai Jiao Tong University School of Medicine	N/A
Formalin-fixed paraffin-embedded colorectal cancer tissues	Renji Hospital affiliated to Shanghai Jiao Tong University School of Medicine	N/A
Chemicals, Peptides, and Recombinant Proteins		
6-cutter restriction enzyme (Hind III)	New England Biolabs	Cat#R0104S
4-cutter restriction enzyme (Dpn II)	New England Biolabs	Cat#R0543S
T4 DNA ligase	Roche	Cat#10799009001
Formaldehyde, 37% (vol/vol)	Merck	Cat#1039992500
PuroDeltatk	BIOCYTOGEN	N/A
pSpCas9(BB)-2A-Puro (Px459)	Addgene	Cat#48139
pGL3-LRF; pGL3-LRR	Addgene	Cat#66936;66937
FuGENE HD	Promega	Cat#E2311
DharmaFECT 1	Thermo Scientific	Cat#T-2001-03
Puromycin Dihydrochloride	Gibco	Cat#A1113802
Azoxymethane (AOM)	Sigma-Aldrich	Cat#A5486
EDTA solution	Invitrogen	Cat#15575020
HEPES (1M)	Gibco	Cat# 15630080

B-27 supplement (50X)	Gibco	Cat# 17504044
N-2 supplement (100X)	Gibco	Cat# 17502001
Recombinant murine epidermal growth factor (EGF)	Gibco	Cat# PMG8043
Recombinant murine Noggin	PeproTech	Cat#25038
Insulin	Gibco	Cat#41400045
bFGF	Gibco	Cat# 13256029
Critical Commercial Assays		
AllPrep DNA/RNA Mini Kit	QIAGEN	Cat#80204
QIAquick Gel Extraction Kit	QIAGEN	Cat#28706
QIAquick PCR Purification Kit	QIAGEN	Cat#28104
Dual-Glo Luciferase Assay System	Promega	Cat#E2940
CelLytic NuCLEAR Extraction Kit	Sigma-Aldrich	Cat#NXTRACT-1KT
LightShift Chemiluminescent EMSA Kit	Thermo Scientific	Cat#20148
Chromatin Immunoprecipitation (ChIP) Assay Kit	Merck Millipore	Cat#17-295
Mouse Tail DNA Mini Kit	Foregene	Cat#DE-05213
Deposited Data		
Raw and analyzed data	This paper	GSE121624
Experimental Models: Cell Lines		
HCT116	ATCC	CCL-247
SW480	ATCC	CCL-228
DLD-1	Kindly provided by Prof. Zou	N/A
Experimental Models: Organisms/Strains		
BALB/c nude mice	Experimental Animal Centre of SIBS	N/A
NOD/Shi-scid/IL-2Rγnull mice	Shanghai Model Organisms Center, Inc	N/A
C57BL/6J mice	Shanghai Model Organisms Center, Inc	N/A
Villin-cre mice	Shanghai Model Organisms Center, Inc	N/A
Sequence-Based Reagents		

Sequences of mRNA siRNAs, see Table S5	Genepharma	N/A
Sequences of sgRNA used in CRC cell lines, see Table S5	BIOCYTOGEN	N/A
Sequences of sgRNA used in mice, see Table S5	Shanghai Model Organisms Center, Inc	N/A
DNA primer sequences, See Table S5	Sangon Biotech	N/A
Software and Algorithms		
PMCA	Genomatix genome analyzer (GGA) software	
ENCODE	Encyclopedia of DNA Elements	https://www.encodeproject.org/
FANTOM5	Functional annotation of the mammalian genome	http://fantom.gsc.riken.jp/5/
CRISPR design tool		http://crispr.mit.edu/
R	R Development Core Team	https://www.r-project.org/
TopHat2	(Trapnell et al., 2009)	http://tophat.cbcb.umd.edu
MACS1.4	(Zhang et al., 2008)	http://liulab.dfci.harvard.edu/MACS/index.html
r3Cseq	(Thongjuea et al., 2013)	https://www.bioconductor.org/
GSVA	(Hanzelmann et al., 2013)	https://www.bioconductor.org/
